# Morpho-physiological analysis of tolerance to aluminum toxicity in rice varieties of North East India

**DOI:** 10.1371/journal.pone.0176357

**Published:** 2017-04-27

**Authors:** Jay Prakash Awasthi, Bedabrata Saha, Preetom Regon, Smita Sahoo, Umakanta Chowra, Amit Pradhan, Anupam Roy, Sanjib Kumar Panda

**Affiliations:** Plant Molecular Biotechnology Laboratory, Department of Life Science and Bioinformatics, Assam University, Silchar, INDIA; National Institute of Plant Genome Research, INDIA

## Abstract

Aluminum (Al) is the third most abundant metal in earth crust, whose chemical form is mainly dependent on soil pH. The most toxic form of Al with respect to plants is Al^3+^, which exists in soil pH <5. Acidic soil significantly limits crop production mainly due to Al^3+^ toxicity worldwide, impacting approximately 50% of the world’s arable land (in North-Eastern India 80% soil are acidic). Al^3+^ toxicity in plants ensues root growth inhibition leading to less nutrient and water uptake impacting crop productivity as a whole. Rice is one of the chief grains which constitutes the staple food of two-third of the world population including India and is not untouched by Al^3+^ toxicity. Al contamination is a critical constraint to plant production in agricultural soils of North East India. 24 indigenous Indica rice varieties (including Badshahbhog as tolerant check and Mashuri as sensitive check) were screened for Al stress tolerance in hydroponic plant growth system. Results show marked difference in growth parameters (relative growth rate, Root tolerance index, fresh and dry weight of root) of rice seedlings due to Al (100 μM) toxicity. Al^3+^ uptake and lipid peroxidation level also increased concomitantly under Al treatment. Histochemical assay were also performed to elucidate uptake of aluminum, loss of membrane integrity and lipid peroxidation, which were found to be more in sensitive genotypes at higher Al concentration. This study revealed that aluminum toxicity is a serious harmful problem for rice crop productivity in acid soil. Based on various parameters studied it’s concluded that Disang is a comparatively tolerant variety whereas Joymati a sensitive variety. Western blot hybridization further strengthened the claim, as it demonstrated more accumulation of Glutathione reductase (GR) protein in Disang rice variety than Joymati under stressed condition. This study also observed that the emergence of lethal toxic symptoms occurs only after 48h irrespective of the dose used in the study.

## Introduction

Aluminum (Al), the third most abundant mineral in earth crust, solubilizes into its most phytotoxic species Al^3+^ in acidic soil from non-toxic Al silicates and oxides [1]. Not only Al, especially in case of rice when the field is flooded due to acidic soil Fe^2+^ increases in the water leading to iron toxicity [2]). 40–50% of the world’s arable soils is acidic which when summed with abundance of Al present in the earth crust leads to Al^3+^ phytotoxicity [3, 4]. Al toxicity is considered to be one of the most serious limiting factor for plant growth in acid soils worldwide [5, 6]. Initial and most dramatic symptom of Al toxicity is rapid inhibition of root elongation, and thus root relative elongation rate has served as a typical marker for level of Al toxicity and tolerance capacity in plants [7]. This effect is caused due to damage in the cells at the root apex which in turn leads to a reduced and stunted root system subsequently impacting the grain quality and plant yield [8, 9]. The symptoms in the roots system like, inhibition of root growth, reduced water and mineral uptake caused by Al^3+^ phytotoxicity starts to appear within minutes after exposure [10].

These preliminary physiological signatures are due to various changes in cellular metabolic activities. One of these important changes in response to Al stress is imbalance in ROS production which induces oxidative stress [11, 12]. This leads to induction of antioxidative defense response cascade which assists to certain extent in containing the oxidative burst [13]. Glutathione reductase (GR) forms a part of the Ascorbate-Glutathione cycle (a part of antioxidative defense mechanism) and thus facilitates the conversion of H_2_O_2_ to H_2_O. An increased GR content in both shoot and root tissues in response to Al stress has been observed to limit the oxidative burst and prevent damage to tissues [14, 13]. Lipid peroxidation is another deleterious process for cellular membrane lipids, for initiation of which ROS molecules are responsible [3]. It proceeds by a free radical chain reaction mechanism and malonialdehyde is one of its end product.

Al-responsive genes have important roles to play towards plant Al-tolerance mechanisms. NRAT1 (Nramp aluminum transporter 1) is a specific Al transporter that is involved in the uptake of Al to cells for sequestration in vacuoles. It is localized in the plasma membrane. The silencing of NRAT1 resulted in decreased Al uptake, increased Al binding to the cell wall, and hence enhanced Al sensitivity. With subsequent detoxification via transport and Al accumulation into cell vacuoles, possibly mediated by *Os*ALS1 (Aluminum sensitive 1) [15, 16]. NRAT1 transporter plays a major role in rice Al tolerance mechanisms [17]

Acidity in surfaces of arable lands can be ameliorated by liming which is quite an arduous approach and not a complete solution to the problem. In response to Al stress plants are found generally to exhibit two type of response, firstly, by exclusion of organic acids from the root apex and secondly, internal tolerance towards Al in the symplast. Plant species, including varieties within a species varies in their response to Al, some are more tolerant to others. Development or screening of genotypes with comparatively greater Al tolerance will go a long way for sustenance of agriculture in acidic soils.

North Eastern (NE) region of India is a biodiversity hotspot and rice is not an exception [18]. For this region rice is the principal staple food crop, accounting for more than 80% of the food grain production [19]. NE region has also diversification in agro-climatic zones demanding for diverse cultivation practices. Rice eco-system in NE include rainfed/irrigated upland, lowland, flood-free and flood-prone, medium land, deep water and hill eco-system [20]. Traditional rice cultivation processes include *Jhum* or shifting cultivation, bun or terrace cultivation and *pani kheti* or wet rice cultivation [21; 22]. Excessive rainfall in NE region is responsible for leaching out of basic cations over a period of time, especially in case of *Jhum* cultivation. Thus paranormal rainfall directing to disturbed soil profile and abundance of Al in soil is exerting its effect on the health of the crop plants. Rice is one of the world’s most important crop, supplying staple food for nearly half of population in the world [23]. Previous reports showed that, rice plants are comparatively more Al-tolerant among small cereal crop species [24, 25, 8] but it is not completely tolerant to Al phytotoxicity. Determining a rice genotype with better Al tolerance capacity relative to others will go a long way in understanding the improved tolerance mechanism in that genotype and development of genotypes with improved traits.

This manuscript report the screening of 24 Indica rice genotypes, including Badshahbhog as tolerant check and Mashuri as sensitive check, for Al tolerance using various physiological, biochemical and histochemical method [26; 27]. The effect of Al on root growth in rice (*Oryza sativa* L.) seedlings, in relation to variations in diverse parameters, to detect the relative Al toxicity tolerance of rice genotypes were studied. This study aims to provide substrate for the study of small RNA mediated gene regulations or any other similar kind of study towards Al stress through identification of contrasting tolerant and sensitive rice genotypes from North East India, a rice agrobiodiversity hotbed. This in turn can pave the way for evolution of Al tolerant rice crop plant through breeding or molecular approaches.

## Materials and methods

### Plant materials and growth condition

Rice genotypes (24 varieties) were collected from the Regional Agriculture Research Station (RARS), Karimganj, Assam, India and Regional Rainfed Lowland Rice Research Station (RRLRRS) Gerua, Guwahati, Assam, India (S1 Table). Adequate amount of viable rice seeds were taken and surface sterilized with 0.1% HgCl_2_ solution for 3–5 minutes with successive shaking. Then, HgCl_2_ solution was discarded, washed thoroughly in tap water for 3–5 minutes, rinsed with distilled water for 2–3 times and decanted. Initially seeds were soaked in water for 12 h, then the seeds were placed properly in petri plates with moisten filter paper and germinated at 28±2°C for 3 days. After three days of incubation the healthy germinated seeds with more or less equal height of root and shoots were transferred into plastic containers (400 ml) containing Hoagland nutritive medium. Plants were grown for a period of 5 days in a growth chamber under white light with photon flux density of 220 μmol m ^-2^ s^−1^ (PAR) over a 14-h photoperiod. After every two days the medium was changed to maintain healthy growth. 5 day old rice seedlings were pretreated with 500 μM CaCl_2_ (pH 4.5) for a day. Pre-treatment solution was then removed and treated with aluminum chloride (AlCl_3_) at different concentration (0, 25, 50 and 100 μM) containing 500 μM CaCl_2_ (pH 4.5) for 12, 24, 48 h. Free Al^3+^ activities in treatment solutions were calculated using GEOCHEM-PC speciation software [28]. 25, 50 and 100 μM corresponds to 7, 14 and 28 μM Al^3+^ activity in solution.

### Measurement of growth and biomass

Growth was measured in term of root length, root fresh weight and dry weight. Ten randomly selected plants of each genotype were selected and the length of the roots was measured by centimeter scale. For Fresh and dry biomass, three plants were randomly grouped for fresh weight measurement, then root tissue was dried at 80°C for 48h and weighed to measure the dry biomass, relative root length rate (RRL), relative tolerance index (RTI) and relative root reduction (RRR%) during Al^3+^ treatment. The whole root lengths (from root-shoot junction to the tip) were measured before and after 12, 24 and 48 h of Al exposure. Each experiment repeated four times.

### Histochemical analyses for Al uptake, lipid peroxidation and plasma membrane integrity

The localization of aluminum was detected with hematoxylin [29, 30]. Roots of intact seedlings were washed in distilled water for 15 min and stained with 0.2% aqueous hematoxylin solution containing 0.02% KIO_3_ for 15 min at room temperature. After washing with distilled water for 15 min, 15 root tips (10 mm) were excised and soaked in 250 ml of 1 M HCl for 1 h. In this staining procedure, aluminum acts as a mordant and causes the binding of hematein (oxidized hematoxylin) to constituents of cells with formation of colored complexes [31].

For histochemical detection of lipid peroxidation [31], roots were stained with 5% Schiff’s reagent [32] for 20 min, which detects aldehydes that originate from lipid peroxides. After the reaction with Schiff’s reagent, roots were rinsed with a sulfite solution (0.5% [w/v] K_2_S_2_O_5_ in 0.05M HCl) for 10 min. The stained roots were kept in the sulfite solution to retain the staining color. The stained roots were then photographed for documentation.

The loss of plasma membrane integrity was evaluated using Evans blue staining method [33] with slight modifications. Roots of intact seedlings were stained with 0.25% (w/v) Evan’s blue in 100μM CaCl_2_ (pH 5.6) for 30 min., then the stained roots were washed with 100μM CaCl_2_ for 15 min. After rinsing with CaCl_2_, root tips were cut for stereoscopic microscope observation.

### Determination of Al uptake and plasma membrane disintegration

Hematoxylin measurement was used for the determination of Al uptake [34] after being stained (described in the previous section) and then homogenised. The homogenate was centrifuged at 13,500 rpm for 10 min and then optical density (OD) of released stain was measured at 490 nm using spectrophotometer (Beckman Coulter DU 730 UV- Vis Spectrophotometer). Aluminum uptake was determined as fold increase in the uptake of dye calculated according to the formula: fold increase = OD of the dye from treated samples/OD of the dye from untreated control samples.

For determination of Evans blue uptake to determine the extent of plasma membrane disintegration, ten previously stained root tip pieces of 10 mm length from the identical positions were placed together and the trapped Evans blue was released by homogenizing the root portions in 1 ml of 1% (w/v) aqueous sodium dodecyl sulphate (SDS) at room temperature. The homogenate was centrifuged at 13,500 rpm for 10 min. The optical density of supernatant was measured spectrophotometrically at 600 nm.

### Lipid peroxidation determination

The level of lipid peroxidation was estimated by the method of [35] in term of MDA content determined by thiobarbituric acid (TBA) reaction. 200mg of fresh tissue was homogenized with 5ml 0.25% TBA containing 10% TCA. The homogenate was boiled for 30 min at 95°C and centrifuged at 10,000g for 10 min. The absorbance of the supernatant was recorded at 532 nm and corrected by subtracting absorbance at 600 nm. The amount of MDA was calculated by using an extinction coefficient of 155 mM^-1^ cm^-1^.

### Measurement of root relative water content (RWC)

Relative Water Content (RWC) was determined by weighing the root and floating it on water (deionized water) for 6h at constant temperature in diffused light. When root became fully turgid, it was reweighed, dried and again weight was determined. RWC was calculated using the formula: RWC = (FW − DW)/(TW − DW) × 100 where FW = Fresh weight, DW = Dry weight and TW = Turgid weight [36].

### RTI measurement

Relative root tolerance index (RTI) was calculated as the maximum root length in Al stress culture divided by the maximum root length in the control [37, 38, 39, 40].

### Germination assay for Al sensitivity in rice genotypes

To determine the sensitivity towards Al an assay was conducted at germination stage using, seeds of 24 rice (5–6 seeds of each variety) varieties were surface sterilized and germinated by soaking in distilled water containing 100 μM concentration of Al at pH 4.5 for 4 days in the dark. Equal amount of seeds were germinated under unstressed condition and root growth was observed [41].

### Al uptake analysis by Atomic Absorption Spectroscopy (AAS)

For determination of Al content, the root were oven dried at 80°C for 72 h. Dried plant material (0.1 g) was acid digested with 5 ml of HCl and nitric acid (HNO_3_) at 120°C until complete digestion was achieved. The final volume was adjusted to 20 ml with deionized water and filter. Then the Al cation content in plant tissues was determined by atomic absorption spectrometer (AA 240, Varian). The absorbance of the standard solutions were measured at 309.5 nm and used to prepare a calibration curve.

### Histochemical detection of H_2_O_2_ and O_2_^-^ in rice leaves

Detection of hydrogen peroxide (H_2_O_2_) was done by 3, 3-diaminobenzidine (DAB) staining [42] and of superoxide radical (O_2_^-^) by NBT staining [43] in leaf segments of both stressed (Al conc. 25, 50, 100μM) and unstressed rice leaf segments. The leaf segments were immersed and infiltrated under vacuum with 1.25 mg/mL DAB staining solution, pH 7.8, dissolved in H_2_O for 6 h, and 3 mg/mL nitro-blue tetrazolium (NBT) staining solution in 10 mM potassium phosphate buffer (pH 7.0) staining solution containing 10 mM NaN_3_ for 30 min at room temperature. Stained leaves were bleached in acetic acid:glycerol:ethanol (1:1:3 v/v) solution at 100°C for 5 min, and stored in glycerol:ethanol (1:4 v/v) solution until photographed.

### Expression analysis

To examine the expression pattern of NRAT1 of the two rice varieties seedlings were exposed to AlCl_3_ concentrations 0 and 100 μM for 24 and 48 hrs. Total RNA was extracted using Macherey-Nagel (Duren, Germany) Nucleospin plant RNA isolation kit following manufacturer’s instruction. One microgram of total RNA was used for first-strand cDNA synthesis using Revert Aid kit (Thermo Scientific, USA) following the manufacturer’s instructions. One-twentieth of the reaction volume was used as template for the PCR amplification of NRAT1 and Actin was used as an internal control (32 cycles). The primer sequences for semi quantitative RT-PCR of NRAT1 were 5′-GAGGCCGTCTGCAGGAGAGG-3′ and 5′-GGAAGTATCTGCAAGCAGCTCTGATGC-3′ [25] the forward and reverse sequences used to amplify Actin were 5’-ATGGCTGACGGCGAGGACATC-3’ and 5’-CAATACCATGCTCGATCGGGTA -3’, respectively.

### Western blot hybridization

Proteins were extracted from 250 mg of leaves of both varieties using an extraction buffer containing 100 mM potassium phosphate buffer (pH 7.0), 1 mM ethylene diamine tetra acetic acid (EDTA), 1% poly vinyl pyrrolidone, (PVP) and protein concentration determined [44]. 30 μg of protein was fractionated on 10% acrylamide gels with sodium dodecyl sulfate (SDS–PAGE) and blotted on to a PVDF membrane by electro transfer blotting unit. Blots were blocked for 2 h at room temperature in 5% blocking buffer (non-fat powdered milk in Tris-buffered saline with 0.1% Tween-20). Rabbit polyclonal antibodies were used at 1/500 dilution in blocking buffer and incubated for overnight at 4°C. The samples were washed three times in TBST (tris buffered saline tween-20) for 5 min each. A secondary goat anti-rabbit (IgG) antibody conjugate to Horse reddish peroxide (HRP) (Santa Cruz, USA) was then used for final detection, at a dilution of 1/1,000. Blots were incubated for 40 min at 4°C, washed 5 times for 5 min each with TBST followed by development in enhanced chemi-luminescence (ECL) substrate solution (Bio-Rad).

### Statistical analysis

For statistical analysis 10–15 seedling per replicate per experiment were taken into account. Statistical comparison between the variances was determined by ANOVA (analysis of variance) and significant differences between mean values (n = 3), where n is the number of times experiments (described in the previous sections) repeated, were determined by Bonferroni analysis. P ≤ 0.05 was deemed to show statistical significance.

The data analysis was carried out using Excel 2013 and statistical software SPSS 20. Each experiment was repeated thrice. Correlation analysis was used to determine the relationships of root length (RL), relative root length (RRL), root tolerance index (RTI), fresh weight (FW), dry weight (DW) relative root reduction (RRR), relative water content (RWC), hematoxylin uptake (HU), evans blue uptake (EBU) and malondialdehyde (MDA) content at 48h after treatment with 0, 25, 50 and 100μM conc. of AlCl_3_.

## Results

Roots of rice genotypes exhibited distinct and visible symptom of aluminum toxicity on short term Al exposure. The primary effect of aluminum toxicity was the reduction in root growth and that’s the reason root physiology parameters were relied upon.

### Measurement of growth

Al stress treatment on rice seedling led to morphological changes and growth inhibition. Growth of rice genotypes as expressed by relative root length were significantly reduced in all varieties. The root length was less reduced in Disang, Swarna sub 1 C, Naveen, Lachit, while reduction was more profound in Joymati, Tulsi Joha, CR Dhan 601, and KMJ-10-1-4 respectively, due to Al treatment for 48h and 100 μM Al concentration compared to other genotypes (Table 1). The relative root length of Al (25, 50, 100 μM) stressed rice roots was found to be decreased over different time frames 12, 24, 48 h but was more marked at 48 h duration. Joymati showed decreased and Disang showed increased relative root length comparable to other varieties across different time frame and Al concentration. The decrease in root elongation was more pronounced in Joymati variety, 31.29% root reduction was observed, while in Disang and Swarna sub 1C variety, only 4.18% and 4.93% reduction respectively. These results indicate higher tolerance of Disang in comparison to other 24 varieties.

**Table 1:** Effect of four aluminum concentrations on relative root length (%) of 24 rice genotypes at 12, 24 and 48 h in the hydroponic assay.

Rice varieties	Al(μM)	Relative root length (%)		
		12h	24h	48h
Disang	0	100.00±1.30	100.00±1.57	100.00±1.85
	25	98.71±1.99	97.35±1.77*	97.01±1.82*
	50	98.18±1.43	96.64±1.79*	96.41±1.22*
	100	97.21±1.38	96.17±1.54*	95.81±1.93*
Swarna sub 1C	0	100.00±1.26	100.00±1.23	100.00±1.42
	25	99.32±1.62	98.22±1.53	97.57±1.43*
	50	98.04±1.45	97.03±1.67	96.23±1.71*
	100	97.12±1.61	96.01±1.20	95.06±1.03*
Naveen	0	100.00±1.40	100.00±1.54	100.00±2.16
	25	99.26±1.52	98.96±1.96	98.88±1.22
	50	96.00±1.42	95.93±1.11	96.48±1.93
	100	95.22±1.38	94.98±1.30	94.84±1.26*
KMJ-6-1-1	0	100.00±1.51	100.00±1.87	100.00±1.77
	25	96.18±1.81	95.69±1.38*	95.55±1.72
	50	95.49±1.86	94.51±1.94*	93.54±1.97*
	100	95.05±1.24	93.68±1.71*	93.13±1.85*
Tapaswini	0	100.00±1.30	100.00±1.50	100.00±1.75
	25	98.34±2.14	97.79±1.02	97.47±1.70
	50	96.86±1.54	96.83±1.92*	95.55±1.34*
	100	93.41±1.51	93.25±1.10*	92.74±1.46*
Badsahbhog	0	100.00±1.98	100.00±1.94	100.00±1.49
	25	98.60±1.96	98.33±2.09	97.13±1.37
	50	97.20±1.70	96.84±1.12	94.74±1.50*
	100	92.91±1.67	92.67±2.02*	92.39±1.51*
Ranjit	0	100.00±1.73	100.00±2.10	100.00±2.15
	25	95.87±1.88	95.79±1.57	95.73±1.39*
	50	94.25±1.90	94.33±1.99	93.99±1.90*
	100	92.83±1.81	92.57±1.88	92.15±1.98*
Lachit	0	100.00±1.76	100.00±1.99	100.00±1.57
	25	95.60±1.88	95.58±1.79	95.36±1.67*
	50	93.14±1.97	93.07±1.74*	92.58±1.55*
	100	92.63±1.76	92.57±1.75*	91.94±1.81*
KMJ-6-1-2	0	100.00±1.90	100.00±1.93	100.00±1.21
	25	98.24±1.59	98.19±1.13	97.64±1.31*
	50	95.49±1.22	95.45±1.51	93.61±1.42*
	100	93.43±1.27	93.39±1.17	91.51±1.38*
Aijung	0	100.00±1.97	100.00±1.81	100.00±1.35
	25	99.77±2.04	99.50±1.72	98.89±1.22
	50	97.85±1.76	96.55±1.54*	97.50±1.73
	100	90.96±1.52*	90.89±1.44*	90.74±1.86*
Kola Joha	0	100.00±1.23	100.00±1.28	100.00±1.21
	25	94.19±1.19	92.98±1.56*	90.15±1.38*
	50	91.39±1.84	90.57±1.24*	89.28±1.99*
	100	90.42±1.23	90.09±1.42*	88.47±1.98*
Sahbhagi Dhan	0	100.00±2.07	100.00±1.10	100.00±1.54
	25	96.44±1.90	96.43±1.43	95.92±1.79
	50	91.66±1.15	91.64±1.65	91.16±1.59
	100	88.46±1.92	88.07±1.52	87.20±1.90
Cauveri	0	100.00±1.48	100.00±1.37	100.00±1.87
	25	99.30±1.40	95.48±1.35*	95.35±1.84*
	50	95.00±1.79	92.95±1.24*	87.44±1.46*
	100	92.50±1.58	87.40±1.44*	86.80±1.57*
Gautam	0	100.00±1.32	100.00±1.75	100.00±1.74
	25	97.67±1.68	96.68±1.54*	96.66±1.27*
	50	97.25±1.95	96.26±1.78*	95.71±2.02*
	100	86.93±1.53	86.88±1.66*	86.64±1.35*
Swarna	0	100.00±1.84	100.00±1.32	100.00±1.91
	25	98.44±1.12	98.43±2.01*	98.41±1.17*
	50	96.82±1.32	96.74±1.21*	96.69±1.48*
	100	86.62±1.34	86.27±1.26*	86.20±1.13*
Kapilee	0	100.00±1.76	100.00±1.57	100.00±1.37
	25	94.78±1.54	94.77±1.43*	93.81±1.94*
	50	94.07±1.71	89.53±1.87*	89.49±2.32*
	100	86.29±1.25*	86.26±1.40*	85.78±1.19*
KMJ -2-1-4	0	100.00±1.47	100.00±1.29	100.00±1.12
	25	97.79±1.90	96.20±1.26	95.63±1.90*
	50	96.39±1.37	91.48±1.82	88.23±1.29*
	100	93.12±1.58	87.80±1.96	85.13±1.76*
Bahadur	0	100.00±1.64	100.00±1.16	100.00±1.94
	25	98.90±1.45	98.87±1.99	98.40±1.59*
	50	93.35±1.81	93.24±1.75*	92.93±1.37*
	100	84.46±1.16	84.17±1.26*	81.77±1.51*
Mashuri	0	100.00±1.61	100.00±1.07	100.00±1.89
	25	90.37±1.32	88.95±1.37*	88.25±1.60*
	50	81.88±2.12	81.64±2.04*	81.55±1.47*
	100	79.96±1.37*	79.49±1.72*	79.26±1.55*
Chandrama	0	100.00±1.23	100.00±1.42	100.00±1.74
	25	96.89±1.56	96.88±1.69	96.79±1.68
	50	79.87±1.63	79.43±1.96*	92.49±1.85*
	100	79.24±1.48*	79.18±1.16*	79.09±1.49*
KMJ-10-1-4	0	100.00±1.02	100.00±1.33	100.00±1.20
	25	95.93±1.45	95.90±2.66*	93.44±1.98*
	50	83.51±1.48*	83.27±1.57*	83.19±2.15*
	100	78.97±1.57*	78.92±1.67*	78.82±1.96*
CR Dhan 601	0	100.00±1.87	100.00±1.65	100.00±1.31
	25	92.98±2.04	91.13±1.35*	89.65±1.63*
	50	82.15±1.17	81.84±1.44*	81.82±1.68*
	100	78.92±1.35*	78.75±1.54*	78.59±1.73*
Tulsi Joha	0	100.00±1.85	100.00±1.48	100.00±1.72
	25	95.40±1.77*	95.27±1.64	94.94±2.10*
	50	81.80±1.68*	81.78±1.98	81.74±1.64*
	100	76.84±1.69*	76.73±1.36*	76.40±1.43*
Joymati	0	100.00±1.42	100.00±1.98	100.00±1.58
	25	95.80±1.71	94.55±1.29	92.81±1.69*
	50	79.41±1.31*	78.49±1.73*	77.18±1.54*
	100	71.34±1.42*	70.36±1.76*	68.70±1.53*

Data presented are mean ± S.E. (n = 10).

Significant mean difference between control and stress plants were significant at *P* < 0.05 (*) by Tukey test.

### Fresh and dry biomass

The presence of Al in the hydroponic medium lowered the root fresh weight and dry weight in all varieties at 24and 48h significantly (p<0.05, S2 Table). It became quite evident that Al phytotoxicity leads to large difference in dry matter accumulation among the cultivars. Genotypes Joymati and Tulsi Joha were more susceptible to Al, since their fresh and dry matter decrease, under stress was much pronounced (35.05%, 41.5% and 44.37%, 57.5%.respectively) at 48h. Of all genotypes, only Disang, Swarna Sub 1C and Lachit had less reduction in root fresh and dry matter accumulation when exposed to Al stress (18.57%, 26.87%, 25.60% and 13.28%, 17.86%, 25.90% respectively) at 48h. The reduction of root fresh weight due to the increase in Al concentration showed a similar trend with that of root dry weight.

### Histochemical detection of hematin, plasma membrane integrity and lipid peroxidation

**Fig 1:**
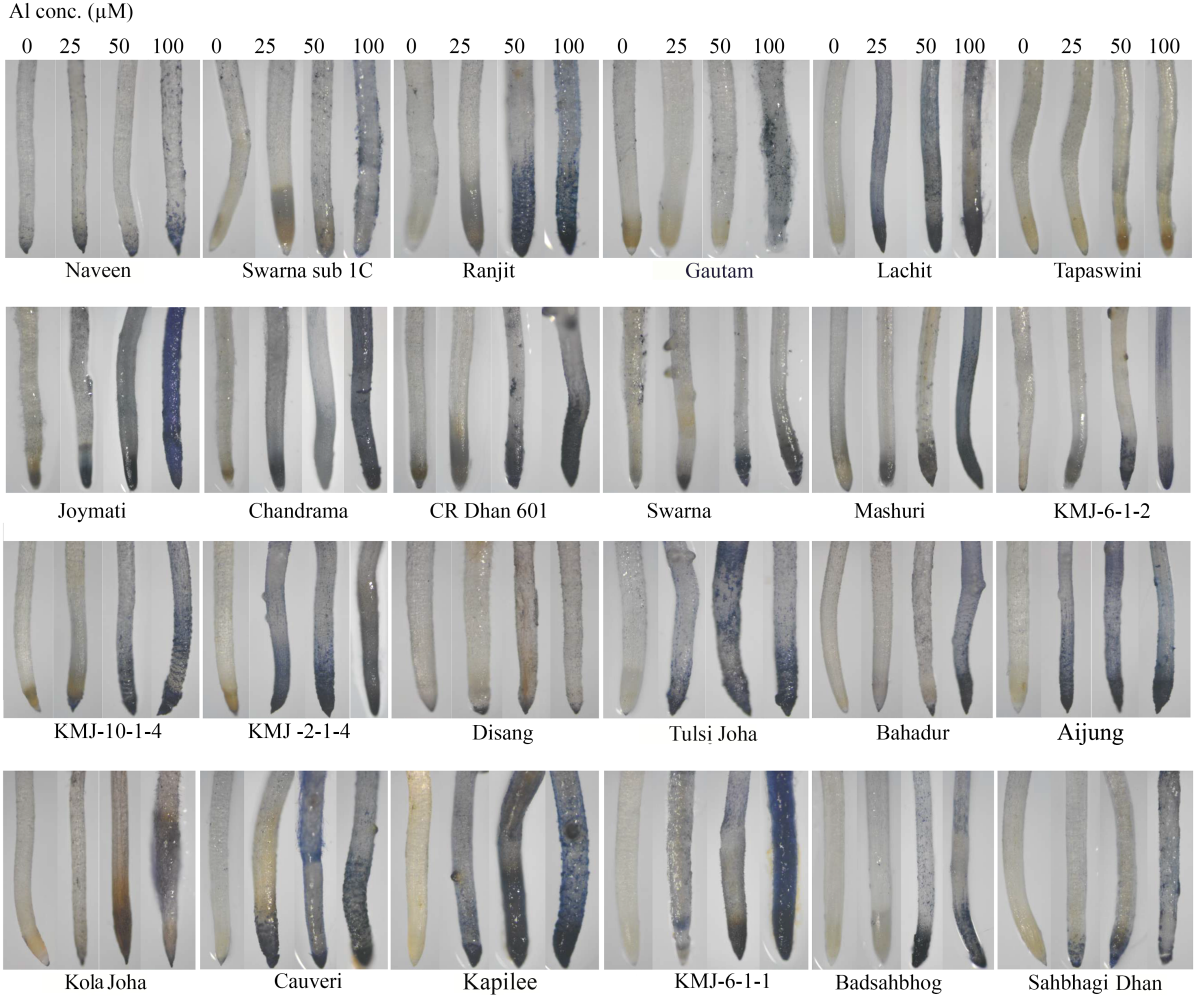
Histochemical detection of aluminum uptake in root tips of rice seedlings. Microscopic views of hematoxylin stained root tips. Intense stained colour represent hematoxylin-Al complexes by the root cells at 48h.

The accumulation of aluminum in plant tissue was observed by staining with hematoxylin (Fig 1) which revealed more accumulation of Al in roots tips treated with 100 μM Al when compared to untreated controls in all rice genotypes. Among twenty four rice genotypes, colour intensity was more in Joymati, Ranjit, Chandrama, Tulsi Joha and Kapilee while lesser intensity was observed in Disang, Tapaswini, Swarna Sub 1C, Kola Joha and Sahbhagi Dhan. Hematoxylin is an Al indicator stain which was useful in assessment of Al localization and accumulation in roots tip, higher concentration of Al in the root tissue caused more intense staining in Joymati than Disang, this response being more pronounced at the root tip. Our result clearly showed the binding of hematin (oxidized hematoxylin, purple bluish color) to indicate the localization of Al in the cytoplasm. The result strongly suggested that Al could readily enter the root cells of rice seedlings.

Loss of membrane integrity and lipid peroxidation, was performed by histochemical staining with Evans blue and Schiff’s reagent. These events were observed exclusively at the root apex from the tip (10mm). The distribution of the histochemical staining patterns of loss of membrane integrity and lipid peroxidation was found in 50 and 100 μM Al treatment (S1 and S2 Figs). These staining patterns were observed on root surface. Uptake of Evans blue dye has been widely used as an indicator of loss of plasma membrane integrity and indicator of cell death. The Evans blue staining indicated that decreased root cell viability under Al Stress, occurred in cells, near the root tips at 48 h. Decreased root cell viability in Joymati, Tulsi Joha, Tapaswini, Ranjit, KMJ-6-1-1 respectively compared to Disang, Sahbhagi Dhan, Bahadur, KMJ-2-1-4 variety during Al stress at 48 h was noticed. Similar results were obtained when the roots of the stressed rice seedlings were treated with Schiff’s reagent (S 4). These results suggest that the loss of membrane integrity and peroxidation of lipids production were caused directly by the interaction of aluminum with the root surface.

### Quantitative determination of Al uptake and plasma membrane disintegration

Spectrophotometric quantification of hematoxylin and Evans blue dye in root tips of control and treated seedlings revealed increased concentration of Al and plasma membrane disintegration in treated seedlings compared to controls (S3 Table), similar to qualitative assay performed by histochemical staining. This was clearly observed by increase in Evans blue uptake by the roots after 24 and 48 h of stress.

Uptake of the dye Evans blue has been widely used as an indicator of loss of plasma membrane integrity as well as an indicator of cell death. In our experiments, nearly 1–3 times fold higher uptake of dye was observed in the root tips of Al- treated seedlings compared to controls (S3 Table). This result indicated a significant uptake of Evans blue and hematoxylin dye in Joymati variety compare to Disang variety at 24 and 48h.

### Measurement of root relative water content

Relative water content acts as an indicator of the water status of a plant as it indicates the absolute amount of water required by plants to reach artificial full saturation. As plants were subjected to Al stress condition, a significant (p<0.05) decline in root relative water content was observed in higher concentration of Al (Table 2). Results on RWC clearly showed that under Al stress condition, reduction was more evident in Joymati, Tulsi Joha, Aijung, Swarna, CR Dhan 601and Gautam respectively whereas less reduction was found in Disang, KMJ-10-1-4 and Swarna sub 1C when compared to other genotypes at 48h.

**Table 2:** Effect of four aluminum concentrations on root relative water content (%) of 24 rice genotypes at 48 h in the hydroponic assay.

Rice varieties Al(μM)	Root Relative water content (%)			
	0	25	50	100
KMJ-10-1-4	97.53±1.84	94.51±2.47	92.03±2.65	89.05±1.03
Disang	93.19±3.20	90.58±4.02	88.18±4.12	86.45±3.32
KMJ -2-1-4	92.72±2.27	89.31±3.32	86.23±2.09	85.46±3.08
KMJ-6-1-2	95.84±0.92	94.14±2.12	89.96±1.71	85.26±1.45*
Kola Joha	94.29±2.04	91.39±1.60	89.99±6.25	84.22±3.79
Mashuri	96.51±1.19	94.27±2.37	92.98±2.54	84.12±2.07*
Swarna sub 1C	92.34±2.97	88.55±3.80	85.30±2.90	83.78±8.09
Badsahbhog	93.01±1.28	90.04±2.70	89.67±2.72	82.24±3.65
KMJ-6-1-1	95.57±1.23	91.78±1.66	86.28±3.18	81.49±3.75*
Sahbhagi Dhan	93.47±2.10	91.39±2.01	89.71±2.48	81.00±2.42*
Tapaswini	93.401±2.40	90.43±3.10	86.20±4.97	80.79±4.08
Chandrama	93.54±1.39	92.58±1.70	85.42±1.04*	80.65±1.47
Kapilee	94.47±2.99	91.43±2.28	90.08±4.12	80.30±5.37*
Naveen	91.34±2.82	86.95±3.86	83.86±5.6	80.25±4.04*
Bahadur	94.49±1.14	86.37±3.00	85.17±3.23	80.19±3.64
Cauveri	95.12±1.68	92.69±1.75	90.40±4.13	79.29±1.02*
Lachit	86.14±4.68	83.85±6.52	81.77±4.65	78.07±3.58
CR Dhan 601	89.83±2.49	86.69±1.60	85.21±1.67	76.85±1.62*
Ranjit	89.11±2.16	86.20±1.85	81.35±2.29	76.74±6.27
Swarna	92.67±4.82	89.29±2.48	82.20±2.51	76.02±6.64*
Tulsi Joha	92.32±3.38	87.96±3.53	82.80±6.87	73.38±7.36*
Gautam	88.06±1.81	80.79±1.59	76.26±3.61	71.53±4.58*
Aijung	86.68±5.45	79.11±1.66	72.91±6.72	71.34±5.66*
Joymati	91.51±2.74	90.11±2.31	84.59±2.40	69.89±2.55*

Data presented are mean ± S.E.(n = 10).

Significant mean difference between control and stress plants were significant at *P* < 0.05 (*) by Tukey test.

### Measurement of lipid peroxidation

Being a product of lipid peroxidation, MDA is generally used as an indicator of extent of lipid peroxidation. MDA is the final product of membrane lipid peroxidation and affects membrane fluidity, cause protein degradation and limits the capacity of ionic transport, which ultimately triggers cell death. MDA content increased in all varieties, 24 and 48 h after Al treatment when compared to respective controls. MDA content in root gradually increased with increase in Al concentrations (S4 Table). Significant (p<0.05) increase of MDA content was observed in Joymati, Tulsi Joha, KMJ-6-1-1, Mashuri, CR Dhan601 and Lachit, while, comparatively lesser increase were found in Disang, swarna sub 1C, Ranjit, Cauveri, Kapilee, Sahbhagi Dhan at higher concentration of Al. In rice roots, significant increase of lipid peroxidation was observed in sensitive genotypes after 24 h and 48h.

### RTI measurement

Relative root tolerance index (RTI) was calculated as the maximum root length in Al stress (100 μM) divided by the maximum root length in the control. Based on RTI, the genotypes were classified into three groups: highly tolerant (≥ 0.90), moderately tolerant (0.80–0.90), and susceptible (≤ 0.80) respectively (S5 Table). The screening was performed in terms of the tolerance index of root growth of these genotypes by Al compared to control. Root growth got significantly (P < 0.05) inhibited by Al in almost all genotypes of rice. Genotypes with tolerance index higher than mean line were considered Al tolerant, whereas genotypes with tolerance index lower than mean line were classified as Al sensitive. Based on this result, the genotypes, viz, Disang, Swarna sub 1 C, Naveen, KMJ-6-1-1, Badsahbhog were considered as tolerant and the genotypes, viz, Joymati, Tulsi Joha, Chandrama, CR Dhan 601, Mashuri, KMJ-10-1-4 as high sensitive to Al (S5 Table).

### Germination assay to determine Al sensitivity

**Fig 2:**
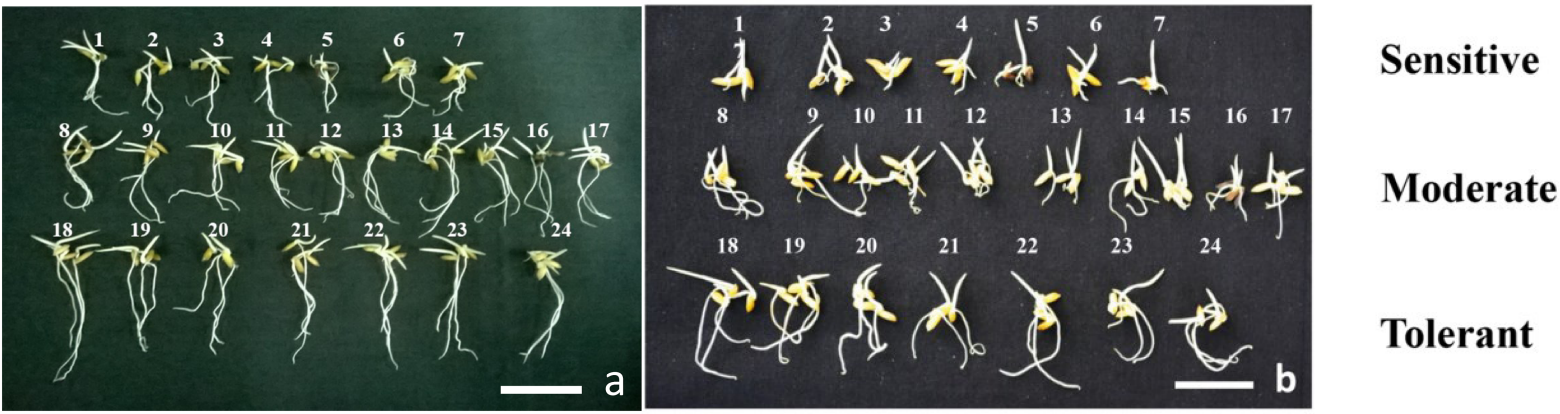
Screening of different 24 rice varieties at 100 μM Al concentration (a) Control; (b) Al stress 100 μM. The numbers denoting different varieties. 1. Joymati, 2. Chandrama, 3. CR Dhan 601, 4. Mashuri, 5. Tulsi Joha, 6. KMJ-10-1-4, 7. Sahbhagi Dhan, 8. Gautam, 9. Swarna, 10. KMJ-2-1-4, 11. Bahadur, 12. Cauveri, 13. Kapilee, 14. Badsahbhog, 15. Aijung, 16. Kola Joha, 17. Tapaswini, 18. Disang, 19. Swarna sub 1 C, 20. Naveen, 21. Ranjit, 22. Lachit, 23. KMJ-6-1-2, 24. KMJ-6-1-1.

100μM Al concentration was used in the germination assay (Fig 2). We tried to screen rice genotypes for more sensitive root growth to Al compared to tolerant genotypes at germination stage. At this condition, root growth in germinating seeds of tolerant genotypes was not significantly affected, while for some genotypes root growth was drastically inhibited (Fig 2b).

### Relationship between root length and relative root reduction (%)

**Fig 3:**
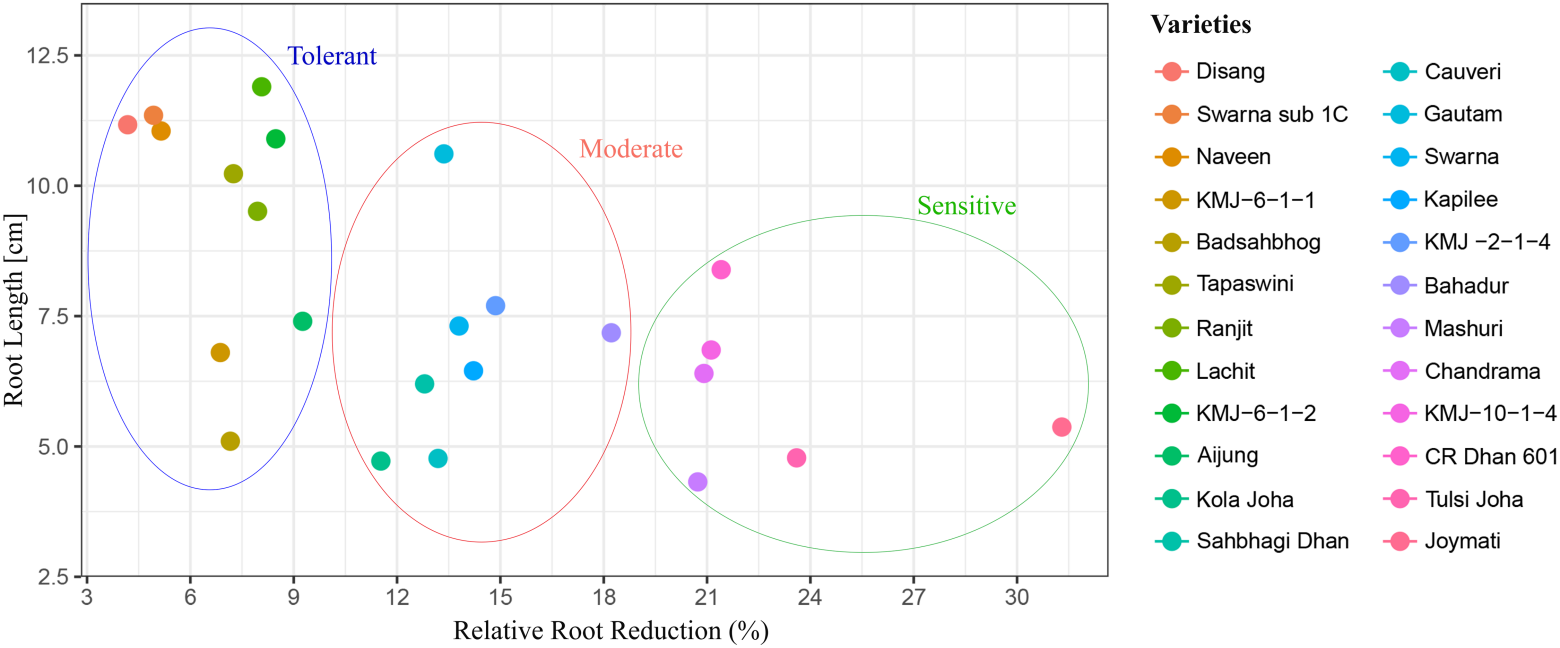
Relationship between root length and relative root reduction (%) of 24 genotypes of rice varieties at 100 μM Al concentration for 48h. The graphical representation was created using ggplot 2 package of R program.

The relationship between root length and relative root length was analyzed independently at 100 μM Al, it revealed that within all genotypes, there was a negative correlation found between root length and relative root reduction (r = 0.34) at higher concentration of Al. (Fig 3). Based on root length, genotypes with less root reduction were classified as tolerant genotypes whereas genotypes with more root reduction were classified as sensitive genotypes (S5 Table)

### Correlation analysis between the screening procedures

The result for correlation analysis (Table 3) clearly depicts the interactions of various parameters in relation to Al stress which corroborate to better understanding of stress responses. The aluminum and stress parameter interactions which was of immediate interest in selection of sensitive and tolerant genotypes was highly significant. The differences among the ten parameter were statistically significant (p <0.05) level. Root length was found to have positive correlation with RRL, RTI, FW, DW and negative correlated with %RRR, RWC, HU, EBU and MDA content (Table 3).

**Table 3:** Correlation between different parameters of 24 rice varieties at 48h.

**Parameter**	**RL**	**RRL**	**RTI**	**%RRR**	**FW**	**DW**	**RWC**	**HU**	**EBU**	**MDA**
**RL**	1	0.431*	0.444*	-0.437*	0.704*	0.736*	-0.05	-0.133	-0.309*	-0.307
**RRL**		1	0.999*	-0.999*	0.626*	0.574*	0.546*	-0.78*	-0.794*	-0.738*
**RTI**			1	-0.999*	0.632*	0.581*	0.539*	-0.776*	-0.789*	0.733*
**%RRR**				1	-0.63*	-0.581*	-0.545*	0.78*	0.795*	0.736*
**FW**					1	0.795*	0.382*	-0.563*	-0.642*	-0.606*
**DW**						1	0.243	-0.434*	-0.534*	-0.472*
**RWC**							1	-0.714*	-0.759*	-0.587*
**HU**								1	0.819*	0.798*
**EBU**									1	0.804*
**MDA**										1

RL, root length; RRL, relative root length; RTI, root tolerance index; RRR%, relative root reduction; FW, fresh weight; DW, dry weight; RWC, relative water content; HU, Hematoxylin uptake; EBU, evanse blue uptake; MDA, Malondialdehyde content. Correlation is significance at the *P < 0.05 level.

### Aluminum contents in tolerant and sensitive genotypes

After preliminary screening, the highly tolerant, moderately tolerant and sensitive genotypes of rice for Al uptake were shortlisted. These results were further verified by Atomic Absorption Spectrometry (AAS) for Al uptake in the shortlisted 7 genotypes. Root Al content of seven genotypes ware estimated at four Al concentration (0, 25, 50, 100 μM) at 48 h. Root Al content (μg/g) of tolerant genotypes (Disang, Swarna sub 1C and Naveen) were significantly lower than those of sensitive genotypes (Joymati and Tulsi Joha), whereas, Cauveri and CR Dhan 601 were found to be moderately tolerant at 25, 50, 100μM Al concentration (Table 4).

**Table 4:** Root Al uptake (μg g^-1^) of seven rice genotypes under four different aluminum concentrations in hydroponic assay at 48 h.

Rice varieties Al(μM)	Root Al uptake (μg/g DW)			
	0	25	50	100
Disang	36.46 ± 13.77	151.04 ± 18.78*	223.95 ± 23.15*	302.08 ± 18.77*
Swrna sub 1C	41.66 ± 18.78	223.96 ± 34.15*	328.13 ± 32.52*	437.5 ± 72.16*
Naveen	41.67 ± 10.41	260.42 ± 37.55*	406.25 ± 18.04*	484.37 ± 9.02*
Cauveri	46.88 ± 9.02	250.00 ± 32.52*	437.50 ± 41.33*	500.00 ± 18.04*
CR Dhan 601	36.46 ± 10.41	354.17 ± 49.68*	416.67 ± 61.40*	541.67 ± 37.55*
Tulsi Joha	52.08 ± 18.77	364.58 ± 10.41*	442.70 ± 13.78*	562.50 ± 9.02*
Joymati	62.50 ± 9.02	458.33 ± 28.99*	572.92 ± 10.71*	630.21 ± 18.77*

Data presented are mean ± S.E (n = 3). Significant mean difference between control and stress plants were significant at *P* < 0.05 (*) by Tukey test.

### Histochemical detection of hydrogen peroxide (H_2_O_2_) and superoxide (O_2_^-^)

**Fig 4:**
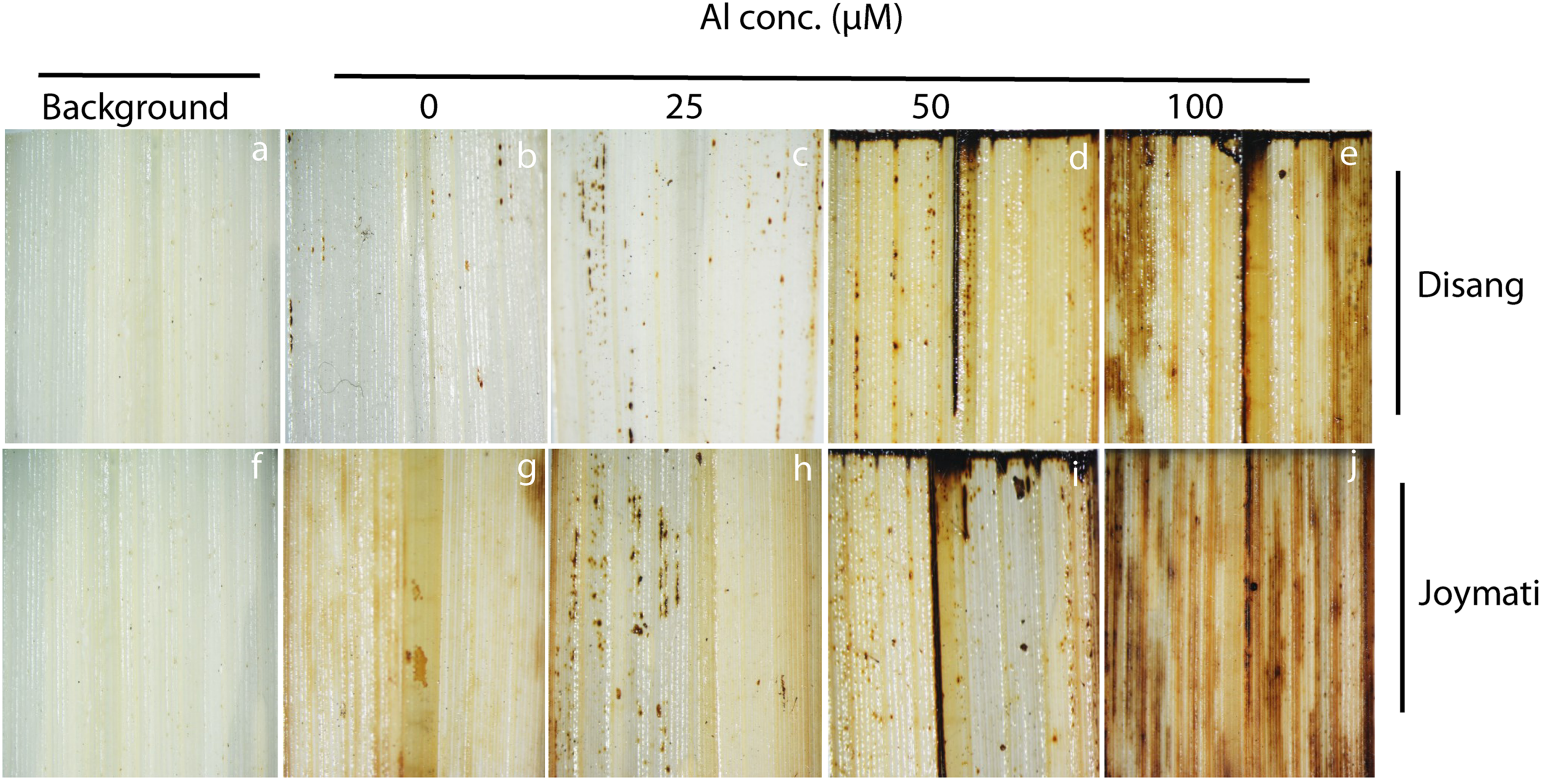
Microscopic view of localization of histochemical detection of hydrogen peroxide *in situ* by DAB uptake method in rice leaves. Leaves of the seedlings grown, containing 0, 25, 50,100 μM Al with 0.5 mM CaCl_2_ pH 4.5. Dark spots represent presence of H_2_O_2_ in rice leaves of Disang and Joymati.

**Fig 5:**
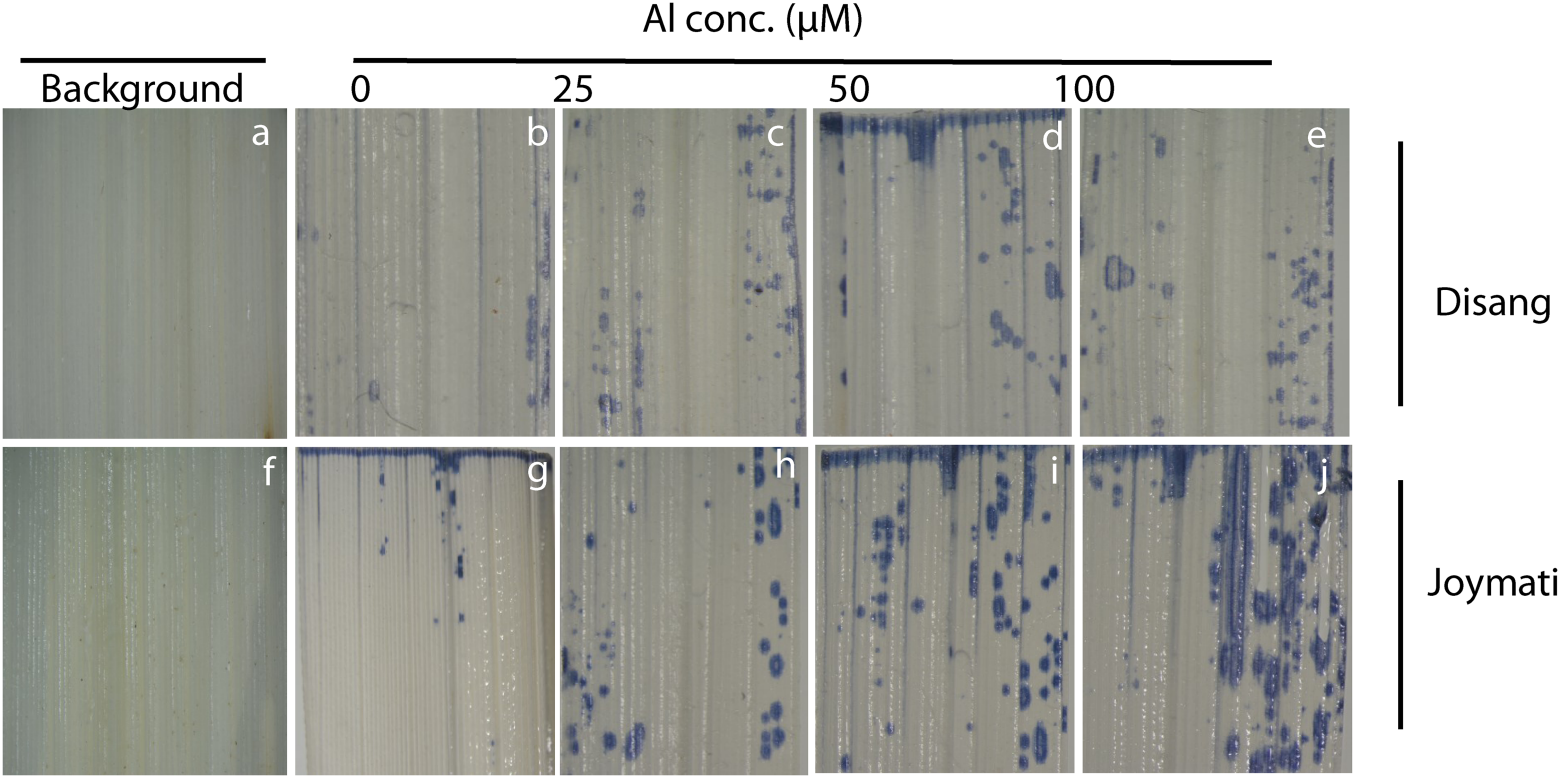
Microscopic view of localization of superoxide anion *in situ* in rice leaves using NBT staining. Leaves of the seedlings grown containing 0, 25, 50,100 μM Al with 0.5 mM CaCl_2_ pH (4.5). Dark stained patches represent O2^•-^ produced in leaves of Disang and Joymati.

H_2_O_2_ and O_2_^-^ production in stressed (Al conc. 25, 50, 100μM) and unstressed rice leaf segments was investigated qualitatively using DAB and NBT histochemical staining respectively (Figs 4 and 5). Under normal physiological conditions, both Joymati (sensitive) and Disang (tolerant) showed low production of O_2_^-^ and H_2_O_2_. However, under Al stress, rice leaf segments of Disang exhibited marked lower NBT and DAB staining particularly at 100 μM than Joymati which is an indication of less ROS production and less oxidative damage in Disang (Figs 4 and 5).

### Expression analysis

**Fig 6:**
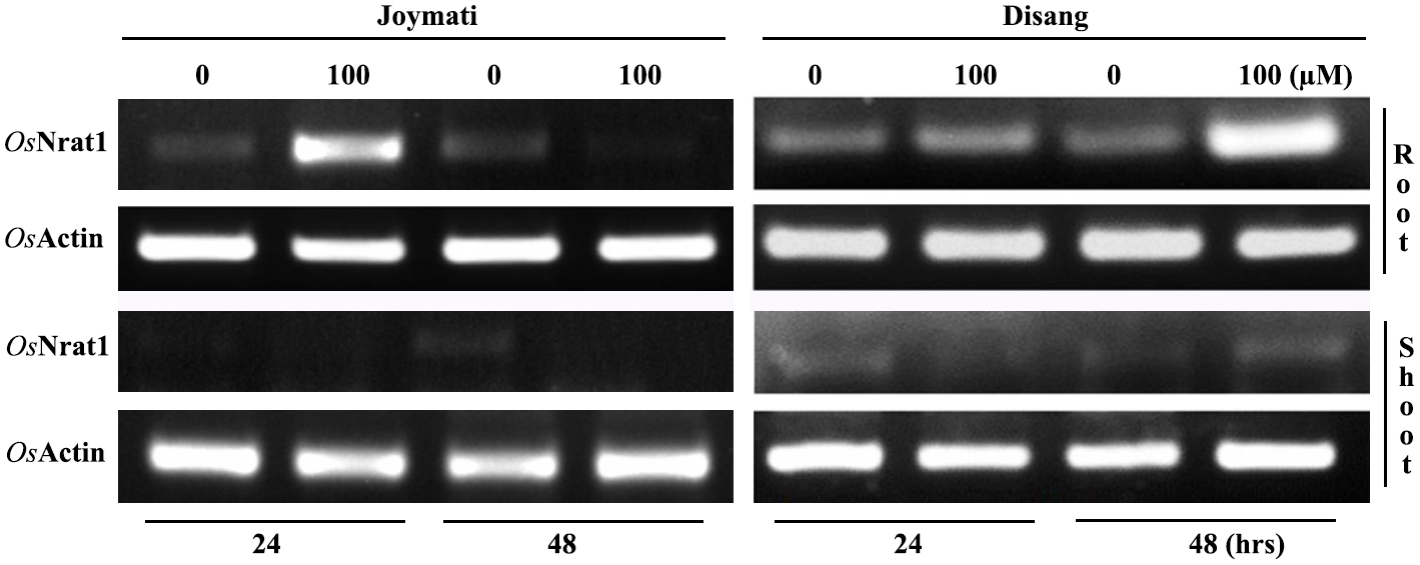
Expression analysis of *Os*NRAT1 under stressed and unstressed condition.

On normalization with Actin, *Os*NRAT1 was found to be induced at 100 μM Al3^+^ in Disang root after both 24 and 48h; the expression was more after 48h of Al treatment as compared to 24h Whereas Joymati showed slightly more expression of NRAT1 in root under 100μM Al^3+^ after 24h, while lesser expression was observed in 48h interval. In shoots negligible expression was observed. (Fig 6).

### Western hybridization of tolerant and sensitive genotypes

**Fig 7:**
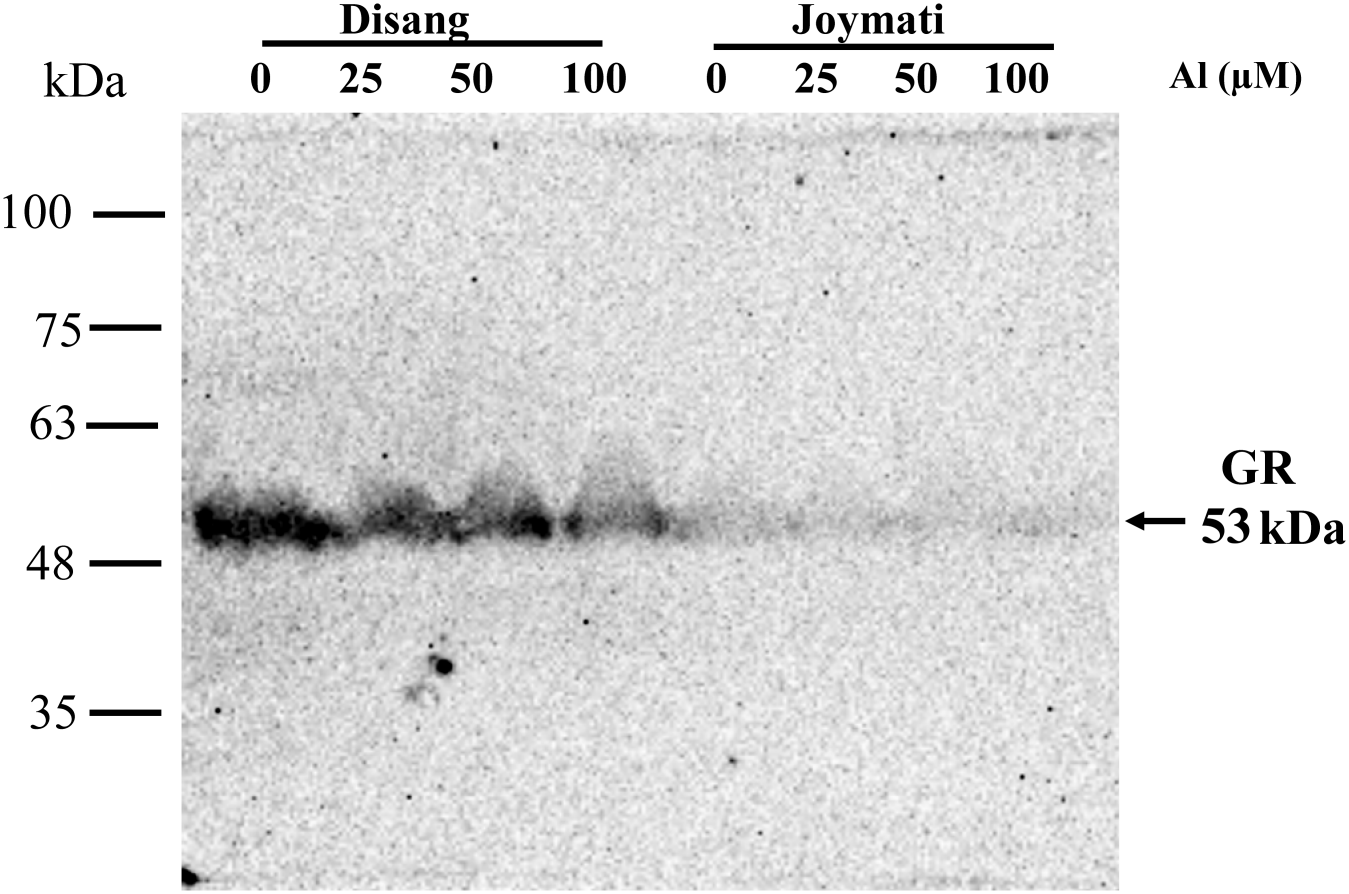
Western blot analysis of rice leaves proteins with glutathione reductases after SDS-Gel electrophoresis. Western blot analysis of Glutathione reductase protein expression in two contrasting rice varieties.

Glutathione reductase (GR) protein expression analysis by Western hybridization was performed for Disang (Tolerant) and Joymati (Sensitive) varieties. The result showed a decrease in the GR protein (53 kDa) levels for both the cultivars across varying concentration of Al, but the more tolerant genotype, Disang showed comparatively more GR content in comparison to Joymati (Fig 7).

## Discussion

Here, extent of Al tolerance in 24 rice genotypes of NE India, a hotspot of rice agrobiodiversity, was investigated. Al phytotoxicity circumscribes plant growth at pH 4.5 stress when observed for 12, 24 and 48 h. Root growth got significantly inhibited in all three Al concentration but most prominent at 100 μM (Table 1). The toxic symptoms observed were very much similar to those reported earlier [5, 6, 26]. Beside root growth inhibition, sharp decrease of fresh and dry biomass (S2 Table) and relative water content of root (Table 2) under Al toxicity were observed. Similar result was recorded in rice [6, 45, 46], pea [47] earlier. Al phytotoxicity has been reported to be responsible for various disturbances at cellular level (disrupts Ca dependent metabolism, lipid peroxidation of plasma membrane), rapid inhibition of cell elongation and division [48]. Al^3+^ can bind to multiple sites of plants, cell wall, plasma membrane, cytoskeleton, and nucleus, effecting their functions [3, 48, 49]. The measurement of Al accumulation was based on hematoxylin staining which has been widely accepted and reported to be helpful in determining the extent of Al accumulation in root [50]. Hematoxylin stains mainly cell wall bound Al [34, 38], the intensity of hematoxylin stain was observed at 48h. Intensity of the dark colour developed due to Al-hematoxylin complex (Hematin), is directly proportional to amount of Al accumulated in the root tissues. Disang variety accumulated far less Al, measured by hematoxylin staining in the root (even when compared to tolerant check Badshahbhog), whereas Joymati accumulated the most (even when compared to sensitive check Mashuri) compared to the other 22 types (Fig 1). Evans blue staining, was exploited as an indicator of cell viability and Schiffs reagents, as a determinant of lipid peroxidation in root cells, to determine the impact of accumulation of Al [29, 31]. There was a clear increase in Evans Blue uptake by the roots after 48 h of Al stress (S1 Fig). Hematoxylin, Evans blue, and Schiffs reagents uptake is apparently showed at 50 μM and 100 μM Al concentration by histochemical (Fig 1; S1 and S2 Figs) and spectrophotometrical analysis (S3 Table). Aluminum treatment caused significant disruption of cell membrane of roots. Similar results were reported for hematoxylin staining in rice [5, 6,25, 46], in barley [51], pea [52], wheat [53], *Medicago truncatula* [7], chickpea [54]; for Evans blue staining in rice [55], in pea [52, 56], maize [57], wheat [58]; and for Schiffs reagents in rice [59], pea [52], tobacco [12], and maize [60].

Lipid peroxidation is a deleterious process in plants [61]. The peroxidation of unsaturated lipids in membrane is the most apparent symptom of oxidative stress. Our result clearly showed that peroxidation of lipid was more found in Joymati variety than Disang variety from all rice genotypes at 48h. MDA being the final product of membrane lipid peroxidation, effects membrane fluidity, causes protein degradation and limits the capacity of ionic transport, which ultimately triggers cell death [52]. Our results showed enhanced malondialdehyde content under Al stress at 24 and 48h (Supplementary 6), similar results were reported in rice [6, 62], Salvinia [63], pea [52], Soyabean [64, 65]. Though all the rice varieties showed lipid peroxidation, the least was observed in Disang and Swarna Sub 1C (S4 Table). Lipid peroxidation is the oxidative degradation of membrane lipids by a free radical chain reaction mechanism that ultimately causes cellular damage, in this case initiated by Al phytoxicity. Disang and Swarna Sub 1C being more resistant to this deleterious effect because of lesser accumulation of Al in the cells as explained earlier by both qualitative and quantitative experiments.

Root length and relative root length were quantified, which showed a negative correlation with root length and relative root reduction at higher concentration of Al (S 8). A similar kind of observation was reported in pigeon pea [66]. Germination assay was performed to check for Al sensitivity at this stage in which Disang genotype was one of the best performing variety (Fig 2). In germination stage of rice, seminal root emerges out from inside of the seed after water absorption and swelling of cells, and then cell division at root apices is needed for root elongation [67]. In this process, water absorption leading to cell expansion seems is a passive process, while cell division requires energy. The hydrolysis of endosperm starch and the supply of oxygen are also needed for development of a seminal root [67]. Seminal roots were found to be reduced in sensitive genotypes, when compared to tolerant genotypes, in presence of Al. Thus root cell expansion and cell division were more in tolerant genotypes. Al-tolerance mechanism at germination stage might be associated with the exclusion of Al from the seminal root apex [41].

Root tolerance index (RTI) has been a suitable marker for the screening of genotypes under Al stress it’s calculated as the maximum root length of the treated sample divided by the maximum root length in the control [38,39,40]. Screening of Rice genotypes for their sensitivity to 100 μM Al was performed in terms of the tolerance index of root of all 24 genotypes on treatment with Al compared to control. Root growth was significantly (p <0.05) inhibited on treatment with Al in all genotypes of rice (Supplementary 7, 8). Determination of correlation coefficients between various characters helps to obtain best combinations of attributes in crops for screening. Correlations between all the ten traits were calculated and were presented in Table 3. The analysis showed significant interaction (p <0.05) among the ten parameters. Similar result observed was in biomass related trait in rice [18, 46], alfalfa [68].

Glutathione reductase is one of the key enzymes of the ascorbate -glutathione cycle that protects cells against oxidative damage and maintains a high GSH/GSSG ratio promoting cellular stability and integrity [69]. In the present study, GR content was estimated in leaf tissue by Western hybridization. Our all previous experiments centered on root tissue being the primary target of Al toxicity but ultimately it also impacts the shoot so to have an overview we used shoot tissue [13, 14] for GR analysis. GR enzyme, involved in the ascorbate–GSH cycle showed a marked decrease in activity in the Al-sensitive and tolerant rice variety Joymati and Disang respectively on Al treatment (Fig 7). But in comparison to Joymati, Disang’s GR content was much more across all Al concentration (Fig 7). Similar kind of result was reported in GR activity of tolerant and sensitive rice varieties by spectrophotometric readings [62]. Histochemical observation was also made for ROS accumulation in Joymati and Disang by NBT and DAB staining which also showed increased accumulation in the sensitive variety Joymati in comparison to Disang (Figs 4 and 5).

To further confirm the physiology shown in the course of Hematoxylin assay, Al uptake analysis of seven (highly tolerant, moderately tolerant and highly sensitive) genotypes were performed by Atomic Absorption Spectroscopy (AAS) after treating the seedlings with 100 μM Al. Al contents was found to be significantly lower for the tolerant (Disang) genotype compare to sensitive (Joymati) genotype (Table 4). In tolerant genotypes, rice plants might be detoxifying certain amount of Al by internal detoxification mechanism (compartmentalization inside vacuoles) and formation of chelate compound with organic acids such as citrate, oxalate [51, 70, 71] which latter exudate out of the cell [72,73,74].

## Conclusions

In this study, some rice genotypes performed better on exposure to Al when compared to tolerant check Badshahbhog. Rice root fresh weight, dry weight relative water content were significantly decreased in sensitive genotypes. Histochemical assays elucidated root apex was more damaged on treatment with higher dosage of aluminum. Al uptake was more profound in Joymati and Tulsi Joha and less in Disang and Swarna sub 1C at 48 h interval after treatment. Based on various parameters our studies revealed that Disang is a comparatively tolerant variety whereas Joymati a sensitive one among the 24 cultivars screened. Being more tolerant Disang genotype can be used to study the mechanism of enhanced tolerance for future use, besides this variety can be used in breeding for transfer of trait to other genotypes. One interesting fact that was observed is the emergence of lethal toxic symptoms after 48h irrespective of the dose used in the study. The identified contrasting varieties Disang and Joymati could further be analyzed to unravel the gene regulations responsible for their opposite behavior to Al stress which could pave the way for genetic engineering or to evolve a truly Al tolerant rice genotype.

## Supporting information

S1 Table Description of rice genotypes. (DOCX)

S2 Table Effect of Al treatment on fresh weight, dry weight in rice roots at 24 and 48h interval. (DOCX)

S3 Table Effect of Al treatment on Evans blue and hematoxylin uptake in rice roots at 24 and 48h interval. (DOCX)

S4 Table Effect of Al treatment on MDA content in rice roots at 24 and 48h interval. (DOCX)

S5 Table Classification of 24 rice genotypes based on root tolerance index (RTI) values at 48h. (DOCX)

S1 Fig Histochemical detection of loss of plasma membrane integrity in root tips of rice seedlings. Microscopic views of Evan’s blue stained in root tips. Intense blue stained root portions represent increased Evans blue uptake at 48h.
(TIF)

S2 Fig Histochemical detection of lipid peroxidation in root tips of rice seedlings. Microscopic views of Schiffs reagents stained of root tips. Intense pinkish colour represent lipid peroxidation by the root cells at 48 h.
(TIF)
