# Supplementary material for: Morpho-physiological analysis of tolerance to aluminum toxicity in rice varieties of North East India
Source: PLoS One. 2017 Apr 27;12(4):e0176357. doi: 10.1371/journal.pone.0176357 (PMC5407633; doi:10.1371/journal.pone.0176357)
Supplement: S1 Table — (DOCX) [file pone.0176357.s001.docx]

**Table S1.** Description of Rice genotypes

| **Varieties** | **Pedigree/ Salient feature** |
| --- | --- |
| Naveen | (Sattari×Jaya) Irrigated Rainfed upland. |
| Swarna sub 1C | Modified Swarna with Sub 1 gene,Flood tolerant,Submergence prone & Shallow low land. |
| Ranjit | (Pankaj × Mahsuri) Recommended for shallow submergence, it is Sali rice variety. |
| Gutam | Rasi mutant, Cold tolerant it is short duration variety, Dwarf, it is Boro rice variety. |
| Lachit | (CRM 13-3241 × Kalinga 2) succeptible to submergence, it is ahu variety. |
| Tapaswini | (Jagannath x Mahsuri) Irrigated medium. |
| Joymati | (Jaya x Mahsuri) Tall,Rainfed it is boro variety, Shallow lowland. |
| Chandrama | Rainfed shallow low land under irrigation it is boro rice variety. |
| CR Dhan 601 | (Jaya×IR 64) Semi-dwarf it is boro rice variety. |
| Swarna | (vasistha× Mahsuri) Rainfed lowland. |
| Mashuri | (Japonica × Indica) Medium late, semi tall it is sutable for raifned lowland areas, |
| KMJ-6-1-2 | Collected from RARS, Karimganj, Assam (Newly bred variety, Parents not yet disclosed) |
| KMJ-10-1-4 | Collected from RARS, Karimganj, Assam (Newly bred variety, Parents not yet disclosed) |
| KMJ -2-1-4 | Collected from RARS, Karimganj, Assam (Newly bred variety, Parents not yet disclosed) |
| Disang | (Lachit×Kalinga-3) Suitable for sowing/planting after recession of flood,it is sali rice variety |
| Tulsi Joha | It is a scented and traditional rice variety. |
| Bahadur | (Pankaj × Mahsuri) Suited to shallow lowland,Rainfed Medium Lands , it is sali rice variety. |
| Aijung | Collected from RRLRRS Gerua, Guwahati, it is traditional variety. |
| Kola Joha | It is a scented and traditional rice variety. |
| Cauveri | (TKM ×TN10) Upland, it is ahu rice variety. |
| Kapilee | (Heera × Ananda) It is Sali rice variety. |
| KMJ-6-1-1 | Collected from RARS, karimganj, Assam (Newly bred variety, Parents not yet disclosed) |
| Badsahbhog | Drought tolerant, aromatic rice variety. |
| Sahbhagi Dhan | Drought prone upland as well as rainfed lowland and direct seeded conditions. It is Sali rice. |
|  |  |

RRLRRS, Gerua- Regional Rainfed Lowland Rice Research station, Gerua, Assam (India).

RARS, Karimganj, Regional Agriculture Research Station, Karimganj, Assam (India).
