# Supplementary material for: Morpho-physiological analysis of tolerance to aluminum toxicity in rice varieties of North East India
Source: PLoS One. 2017 Apr 27;12(4):e0176357. doi: 10.1371/journal.pone.0176357 (PMC5407633; doi:10.1371/journal.pone.0176357)
Supplement: S2 Table — (DOCX) [file pone.0176357.s002.docx]

| **Varieties** | **Al Conc.(µM)** | **Fresh Weight(mg)** | | **Dry Weight(mg)** | |
| --- | --- | --- | --- | --- | --- |
|  |  | **24 h** | **48h** | **24 h** | **48h** |
| Disang | 0 | 88.60±0.91 | 89.92±3.22 | 7.41±0.22 | 7.98±0.45 |
|  | 25 | 82.51±2.56 | 85.70±2.98 | 7.05±0.27 | 7.84±0.50 |
|  | 50 | 79.02±1.42 | 80.18±4.81 | 6.95±0.07 | 7.18±0.58 |
|  | 100 | 72.34±1.91* | 73.22±1.85* | 6.71±0.10 | 6.92±0.07 |
| Swarna sub 1C | 0 | 82.55±4.12 | 87.95±1.57 | 12.45±1.65 | 12.51±0.35 |
|  | 25 | 79.30±6.57 | 80.40±3.24* | 11.65±0.29 | 11.70±1.78 |
|  | 50 | 71.86±2.25 | 73.72±1.02* | 11.13±0.22 | 11.16±0.42 |
|  | 100 | 62.30±2.99* | 64.31±0.79* | 10.10±0.92 | 10.28±1.80 |
| Naveen | 0 | 89.25±3.43 | 92.31±2.00 | 12.65±0.46 | 12.77±0.93 |
|  | 25 | 79.85±5.79 | 85.36±3.84 | 11.32±1.07 | 11.40±2.34 |
|  | 50 | 62.60±0.96* | 78.45±0.07* | 10.98±0.70 | 11.06±1.01 |
|  | 100 | 54.20±1.21* | 56.60±0.40* | 10.20±1.20* | 10.32±1.63 |
| KMJ-6-1-1 | 0 | 63.75±1.55 | 65.31±0.40 | 9.33±0.88 | 9.53±0.69 |
|  | 25 | 49.33±0.88* | 50.33±0.88 | 7.98±0.55 | 8.05±0.42 |
|  | 50 | 37.0±1.52* | 46.92±0.33* | 6.86±0.50 | 6.92±0.57 |
|  | 100 | 27.66±1.76* | 32.89±0.70* | 6.32±1.03* | 6.46±0.93* |
| Tapaswini | 0 | 48.55±0.78 | 66.00±2.29 | 6.80±0.26 | 9.05±0.27 |
|  | 25 | 47.65±0.80 | 59.01±4.97 | 5.05±0.27* | 8.35±0.52 |
|  | 50 | 42.50±1.30 | 48.37±2.10* | 4.15±0.10* | 7.36±0.69 |
|  | 100 | 35.62±3.79* | 41.87±2.59* | 3.95±0.11* | 5.95±0.60* |
| Badsahbhog | 0 | 64.88±4.24 | 66.23±0.74 | 5.00±0.057 | 6.92±0.36 |
|  | 25 | 60.01±4.40 | 61.33±1.59 | 3.90±0.11 | 6.71±0.90 |
|  | 50 | 55.92±0.68 | 58.23±3.19* | 3.53±0.20 | 6.09±0.057 |
|  | 100 | 44.55±2.22* | 45.10±1.06* | 3.30±0.45* | 5.39±0.71* |
| Ranjit | 0 | 69.60±0.34 | 75.50±5.60 | 9.85±1.00 | 10.13±1.55 |
|  | 25 | 63.50±3.28 | 65.65±1.06 | 8.50±1.00 | 8.96±0.68 |
|  | 50 | 60.90±0.61 | 61.90±1.49 | 7.80±1.50 | 8.10±1.53 |
|  | 100 | 52.95±0.47* | 53.41±2.09* | 7.15±0.48* | 7.91±1.04* |
| Lachit | 0 | 82.10±2.48 | 86.5±4.22 | 13.20±0.55 | 13.24±0.60 |
|  | 25 | 76.32±4.99 | 79.30±5.62 | 11.85±1.52 | 11.88±0.74 |
|  | 50 | 68.0±3.46 | 69.55±0.66* | 10.45±0.23 | 10.49±0.55* |
|  | 100 | 62.30±2.59* | 64.35±1.08* | 9.86±0.86* | 9.81±0.52* |
| KMJ-6-1-2 | 0 | 80.56±7.09 | 81.11±4.15 | 8.74±0.23 | 8.94±1.02 |
|  | 25 | 76.80±2.93 | 77.32±2.17 | 7.92±0.14 | 8.05±1.10 |
|  | 50 | 70.50±0.75 | 72.14±6.77 | 7.02±0.12 | 7.28±1.03 |
|  | 100 | 46.50±3.90* | 48.98±2.81* | 6.23±0.44 | 6.56±0.81 |

**Table. S2** Effect of Al treatment on fresh weight, dry weight in rice roots at 24 and 48h interval.

Continued……

| Aijung | 0 | 57.35±1.36 | 61.23±1.24 | 5.63±0.20 | 6.58±0.45 |
| --- | --- | --- | --- | --- | --- |
|  | 25 | 49.56±2.41* | 52.80±1.63 | 4.90±0.32 | 5.43±0.24 |
|  | 50 | 42.66±0.88* | 43.05±2.65* | 4.43±0.12* | 5.09±0.41 |
|  | 100 | 34.33±1.20* | 36.82±2.57* | 3.33±0.32 | 4.52±0.39 |
| Kola Joha | 0 | 58.0±0.80 | 59.81±1.04 | 5.53±0.16 | 5.86±0.48 |
|  | 25 | 52.3.±1.67 | 53.69±1.78 | 4.85±0.14 | 5.11±0.84 |
|  | 50 | 46.0±1.27 | 47.12±0.85 | 3.56±0.37 | 4.87±0.77 |
|  | 100 | 36.3±1.93 | 38.11±2.52 | 3.04±0.96 | 4.05±0.50 |
| Sahbhagi Dhan | 0 | 48.33±2.84 | 50.23±1.66 | 5.30±0.23 | 6.23±0.57 |
|  | 25 | 38.33±1.76* | 39.41±2.09* | 4.70±0.11 | 5.60±0.40 |
|  | 50 | 34.66±1.33* | 36.21±0.95* | 3.76±0.08* | 4.96±0.12 |
|  | 100 | 28.0±0.57* | 30.30±1.73* | 3.50±0.40* | 4.10±0.32* |
| Cauveri | 0 | 54.23±0.92 | 55.42±1.80 | 7.89±0.38 | 8.56±0.53 |
|  | 25 | 47.36±1.15* | 48.13±0.49 | 7.00±0.57 | 7.96±0.90 |
|  | 50 | 41.20±1.58* | 43.78±0.81 | 6.00±0.58 | 7.13±1.11 |
|  | 100 | 32.67±1.94* | 34.84±0.48* | 4.66±0.67* | 6.00±0.57 |
| Gautam | 0 | 80.32±4.65 | 89.70±0.41 | 10.85±0.07 | 10.92±0.37 |
|  | 25 | 76.75±0.94 | 76.80±1.51* | 10.20±1.19 | 10.23±0.39 |
|  | 50 | 64.5±1.80* | 68.50±1.71* | 9.45±0.23 | 9.51±0.96 |
|  | 100 | 44.75±1.03* | 47.20±0.55* | 8.40±0.80 | 8.53±0.69 |
| Swarna | 0 | 72.5670.70 | 75.74±2.15 | 10.66±0.46 | 10.84±0.71 |
|  | 25 | 68.83±0.92 | 60.66±0.88* | 9.16±0.44 | 9.25±0.65 |
|  | 50 | 49.81±2.40* | 58.66±3.17* | 7.30±0.35* | 7.46±0.31* |
|  | 100 | 43.21±1.76* | 49.00±2.50* | 6.90±0.34* | 7.01±0.28* |
| Kapilee | 0 | 72.35±1.15 | 75.10±1.01 | 8.47±0.23 | 9.23±0.34 |
|  | 25 | 68.43±4.07 | 69.46±0.57 | 8.00±0.57 | 8.91±0.42 |
|  | 50 | 64.66±3.08 | 65.13±1.55 | 7.00±1.00 | 7.86±0.31 |
|  | 100 | 55.0±2.30* | 56.91±1.72* | 5.33±0.33* | 6.03±0.20* |
| KMJ -2-1-4 | 0 | 78.30±1.97 | 79.08±0.82 | 9.23±0.60 | 9.85±0.56 |
|  | 25 | 68.42±1.85 | 70.59±4.30 | 8.01±0.13 | 8.26±0.32 |
|  | 50 | 56.01±2.17 | 58.76±8.73 | 7.89±0.93 | 7.91±1.03 |
|  | 100 | 49.37±1.84* | 51.79±4.22* | 7.14±0.52 | 7.22±0.16 |
| Bahadur | 0 | 78.23±0.84 | 80.56±0.75 | 10.32±0.52 | 10.50±0.37 |
|  | 25 | 72.31±3.69 | 74.22±1.56* | 9.10±1.04 | 9.51±0.55 |
|  | 50 | 53.66±2.60* | 56.30±1.84* | 7.33±0.33 | 7.63±0.47* |
|  | 100 | 41.32±1.58* | 45.86±1.26* | 6.58±0.86* | 6.72±0.54* |
| Mashuri | 0 | 48.66±3.17 | 59.66±2.02 | 5.90±0.23 | 6.36±0.46 |
|  | 25 | 41.43±0.29 | 49.33±0.88* | 5.03±0.44 | 5.40±0.45 |
|  | 50 | 36.03±0.99* | 38.00±2.10* | 4.06±0.35* | 4.83±0.41 |
|  | 100 | 21.70±1.80* | 30.66±1.20* | 2.86±0.34* | 4.10±0.05 |

Continued……

| Chandrama | 0 | 60.05±1.42 | 68.35±3.35 | 7.55±0.16 | 8.12±0.50 |
| --- | --- | --- | --- | --- | --- |
|  | 25 | 56.05±1.18 | 61.02±9.22 | 6.55±0.17 | 6.90±0.55 |
|  | 50 | 46.50±2.36* | 55.30±5.86* | 5.62±0.56* | 6.12±0.58 |
|  | 100 | 38.00±1.25* | 42.86±2.86* | 4.55±0.029* | 5.81±0.52 |
| KMJ-10-1-4 | 0 | 58.23±5.13 | 61.81±0.32 | 9.95±0.59 | 9.35±1.87 |
|  | 25 | 47.65±2.20 | 49.93±0.16* | 8.35±0.97 | 7.71±1.29 |
|  | 50 | 43.50±2.44* | 44.21±1.58* | 8.12±1.33 | 7.41±1.68 |
|  | 100 | 39.51±1.92* | 40.00±2.08* | 7.53±0.66 | 5.66±0.15* |
| CR Dhan 601 | 0 | 84.50±0.76 | 85.15±2.97 | 8.50±0.49 | 9.16±0.64 |
|  | 25 | 75.38±5.44 | 76.08±6.47 | 6.65±0.15* | 7.90±0.91 |
|  | 50 | 62.86±4.73* | 67.00±1.52* | 5.12±0.17* | 6.32±0.37 |
|  | 100 | 50.40±4.16* | 53.14±2.11* | 4.93±0.53* | 5.48±0.51* |
| Tulsi Joha | 0 | 54.21±3.24 | 67.55±2.81 | 7.13±0.44 | 7.56±1.16 |
|  | 25 | 39.60±1.15* | 53.90±1.41* | 6.45±0.07 | 6.81±0.86 |
|  | 50 | 33.60±3.07* | 45.15±2.07* | 6.02±0.92 | 6.22±1.14 |
|  | 100 | 29.60±2.25* | 41.50±1.89* | 4.10±0.31* | 4.52±0.98 |
| Joymati | 0 | 57.85±3.68 | 74.40±1.36 | 8.45±0.29 | 8.62±0.64 |
|  | 25 | 49.56±4.84 | 54.20±2.10* | 7.36±0.91 | 7.51±0.80 |
|  | 50 | 41.30±4.35 | 44.12±3.87* | 5.55±1.03 | 5.63±0.98 |
|  | 100 | 31.0±2.08* | 35.05±1.55* | 3.75±0.25* | 4.05±0.27* |

Data presented are mean ± S.E.(n=10).

Significant mean difference between control and stress plants were significant at *P* < 0.05 (*) by Tukey test.
