# Supplementary material for: Morpho-physiological analysis of tolerance to aluminum toxicity in rice varieties of North East India
Source: PLoS One. 2017 Apr 27;12(4):e0176357. doi: 10.1371/journal.pone.0176357 (PMC5407633; doi:10.1371/journal.pone.0176357)
Supplement: S3 Table — (DOCX) [file pone.0176357.s003.docx]

**Table. S5** Effect of Al treatment on Evans blue and heamatoxylin uptake in rice roots at 24 and 48h interval.

|  |  | **Fold increase/uptake** | | | |
| --- | --- | --- | --- | --- | --- |
| **Varieties** | **Al Conc.(µM)** | **Evans Blue** | | **Heamatoxylin** | |
|  |  | **24h** | **48h** | **24h** | **48h** |
| Disang | 0 | 1.00±0.034 | 1.00±0.043 | 1.00±0.023 | 1.00±0.023 |
|  | 25 | 1.20±0.044* | 1.19±0.053* | 1.09±0.027 | 1.09±0.022 |
|  | 50 | 1.32±0.041* | 1.58±0.055* | 1.12±0.018 | 1.42±0.031 |
|  | 100 | 1.53±0.045* | 1.89±0.054* | 1.18±0.014* | 1.67±0.022* |
| Swarna sub 1C | 0 | 1.00±0.054 | 1.00±0.044 | 1.00±0.198 | 1.00±0.161 |
|  | 25 | 1.11±0.053 | 1.11±0.041* | 1.06±0.197 | 1.36±0.154* |
|  | 50 | 1.31±0.050* | 1.41±0.052* | 1.21±0.042* | 1.77±0.152* |
|  | 100 | 1.61±0.052* | 1.76±0.046* | 1.45±0.047* | 1.90±0.147* |
| Naveen | 0 | 1.00±0.043 | 1.00±0.048 | 1.00± 0.041 | 1.00±0.055 |
|  | 25 | 1.16±0.056* | 1.35±0.046* | 1.06± 0.052 | 1.66±0.041* |
|  | 50 | 1.56±0.054* | 1.58±0.045* | 1.40±0.054* | 1.98±0.045* |
|  | 100 | 1.69±0.051* | 2.14±0.069* | 1.54±0.056* | 1.94±0.024* |
| KMJ-6-1-1 | 0 | 1.00±0.032 | 1.00±0.044 | 1.00±0.101 | 1.00±0.047 |
|  | 25 | 1.10±0.033* | 1.45±0.055* | 1.05±0.082 | 1.29±0.059 |
|  | 50 | 1.33±0.043* | 1.85±0.054* | 1.19±0.098 | 1.47±0.069* |
|  | 100 | 1.81±0.080* | 2.03±0.050* | 1.34±0.102* | 1.78±0.102* |
| Tapaswini | 0 | 1.00±0.038 | 1.00±0.051 | 1.00±0.016 | 1.00±0.039 |
|  | 25 | 1.65±0.060* | 1.40±0.039* | 1.65±0.025* | 1.42±0.053* |
|  | 50 | 1.78±0.059* | 1.33±0.080* | 1.92±0.025* | 2.35±0.054* |
|  | 100 | 1.83±0.056* | 2.00±0.046* | 2.07±0.027* | 2.48±0.056* |
| Badsahbhog | 0 | 1.00±0.042 | 1.00±0.053 | 1.00±0.033 | 1.00±0.047 |
|  | 25 | 1.37±0.054* | 1.23±0.056* | 1.18±0.053* | 1.13±0.056* |
|  | 50 | 1.53±0.046* | 1.41±0.045* | 1.31±0.051* | 1.65±0.060* |
|  | 100 | 1.63±0.048* | 1.86±0.035* | 1.44±0.056* | 1.96±0.077* |
| Ranjit | 0 | 1.00±0.048 | 1.00±0.044 | 1.00±0.158 | 1.00±0.035 |
|  | 25 | 1.13±0.055* | 1.25±0.049* | 1.14±0.170 | 1.37±0.041 |
|  | 50 | 1.44±0.063* | 1.60±0.071* | 1.48±0.204 | 1.89±0.043 |
|  | 100 | 1.65±0.069* | 2.01±0.043* | 1.77±0.159 | 1.96±0.040 |
| Lachit | 0 | 1.00±0.040 | 1.00±0.038 | 1.00±0.040 | 1.00±0.039 |
|  | 25 | 1.18±0.036 | 1.27±0.055 | 1.62±0.041 | 1.56±0.057 |
|  | 50 | 1.54±0.065* | 1.69±0.042* | 1.88±0.044* | 2.05±0.058* |
|  | 100 | 1.70±0.066* | 1.86±0.044* | 1.99±0.047* | 2.38±0.045* |
| KMJ-6-1-2 | 0 | 1.00±0.051 | 1.00±0.054 | 1.00±0.043 | 1.00±0.038 |
|  | 25 | 1.15±0.053 | 1.27±0.046* | 1.09±0.054 | 1.52±0.037 |
|  | 50 | 1.38±0.071* | 1.53±0.051* | 1.24±0.055* | 2.1470.048* |
|  | 100 | 1.71±0.069* | 2.03±0.077* | 1.54±0.070* | 2.32±0.059* |

Continued….

| Aijung | 0 | 1.00±0.039 | 1.00±0.035 | 1.00±0.055 | 1.00±0.032 |
| --- | --- | --- | --- | --- | --- |
|  | 25 | 1.16±0.041* | 1.58±0.057* | 1.25±0.042* | 1.61±0.045* |
|  | 50 | 1.38±0.052* | 2.10±0.048* | 1.51±0.060* | 1.78±0.046* |
|  | 100 | 1.74±0.063* | 2.46±0.042* | 1.71±0.051* | 1.94±0.046* |
| Kola Joha | 0 | 1.00±0.043 | 1.00±0.038 | 1.00±0.069 | 1.00±0.032 |
|  | 25 | 1.26±0.048* | 1.58±0.043* | 1.12±0.068* | 1.36±0.045* |
|  | 50 | 1.47±0.047* | 1.70±0.045* | 1.48±0.051* | 1.75±0.046* |
|  | 100 | 1.76±0.054* | 2.01±0.053* | 1.69±0.038* | 1.97±0.046* |
| Sahbhagi Dhan | 0 | 1.00±0.041 | 1.00±0.038 | 1.00±0.038 | 1.00±0.033 |
|  | 25 | 1.19±0.046* | 1.48±0.032* | 1.30±0.020* | 1.30±0.041* |
|  | 50 | 1.4670.051* | 1.68±0.032* | 1.54±0.061* | 1.64±0.049* |
|  | 100 | 1.60±0.055* | 1.98±0.037* | 1.63±0.059* | 1.73±0.051* |
| Cauveri | 0 | 1.00±0.049 | 1.00±0.037 | 1.00±0.070 | 1.00±0.029 |
|  | 25 | 1.25±0.054* | 1.57±0.035* | 1.16±0.082* | 1.46±0.054* |
|  | 50 | 1.50±0.058* | 1.83±0.040* | 1.23±0.083* | 1.72±0.046* |
|  | 100 | 1.66±0.088* | 2.29±0.049* | 1.40±0.081* | 1.86±0.042* |
| Gautam | 0 | 1.00±0.040 | 1.00±0.033 | 1.00±0.128 | 1.00±0.079 |
|  | 25 | 1.03±0.042 | 1.41±0.051 | 1.36±0.061 | 1.53±0.080 |
|  | 50 | 1.56±0.066 | 1.89±0.086 | 1.67±0.054* | 1.78±0.089* |
|  | 100 | 1.90±0.079* | 2.33±0.119* | 1.71±0.112* | 2.08±0.091* |
| Swarna | 0 | 1.00±0.065 | 1.00±0.043 | 1.00±0.059 | 1.00±0.044 |
|  | 25 | 1.06±0.056 | 1.36±0.055* | 1.39±0.108* | 1.68±0.039* |
|  | 50 | 1.37±0.067* | 2.19±0.055* | 1.56±0.114* | 2.52±0.050* |
|  | 100 | 1.68±0.066* | 2.26±0.046* | 1.86±0.112* | 2.63±0.048* |
| Kapilee | 0 | 1.00±0.036 | 1.00±0.049 | 1.00±0.159 | 1.00±0.075 |
|  | 25 | 1.30±0.040* | 1.69±0.039* | 1.03±0.194 | 1.17±0.101* |
|  | 50 | 1.46±0.030* | 1.89±0.036* | 1.14±0.179 | 1.44±0.080* |
|  | 100 | 1.74±0.042* | 2.18±0.040* | 1.21±0.175* | 1.98±0.110* |
| KMJ -2-1-4 | 0 | 1.00±0.040 | 1.00±0.046 | 1.00±0.040 | 1.00±0.041 |
|  | 25 | 1.18±0.041* | 1.21±0.042* | 1.29±0.054* | 1.51±0.034* |
|  | 50 | 1.47±0.039* | 1.39±0.040* | 1.71±0.053* | 2.10±0.049* |
|  | 100 | 1.89±0.046* | 1.96±0.066* | 1.89±0.061* | 2.39±0.053* |
| Bahadur | 0 | 1.00±0.025 | 1.00±0.037 | 1.00±0.043 | 1.00±0.073 |
|  | 25 | 1.14±0.027* | 1.37±0.038* | 1.24±0.067* | 1.10±0.022 |
|  | 50 | 1.59±0.037* | 1.74±0.037* | 1.61±0.040* | 1.86±0.038* |
|  | 100 | 1.79±0.055* | 1.97±0.046* | 1.87±0.037* | 1.99±0.054* |
| Mashuri | 0 | 1.00±0.036 | 1.00±0.035 | 1.00±0.024 | 1.00±0.029 |
|  | 25 | 1.06±0.034 | 1.40±0.026* | 1.26±0.053* | 1.26±0.014* |
|  | 50 | 1.42±0.066* | 1.60±0.0445* | 1.52±0.048* | 1.96±0.056* |
|  | 100 | 1.68±0.079* | 1.84±0.060* | 1.85±0.043* | 2.28±0.050* |

Continued….

| Chandrama | 0 | 1.00±0.035 | 1.00±0.069 | 1.00±0.019 | 1.00±0.020 |
| --- | --- | --- | --- | --- | --- |
|  | 25 | 1.47±0.087* | 1.60±0.061* | 1.16±0.018* | 1.44±0.027* |
|  | 50 | 1.52±0.078* | 1.78±0.058* | 1.67±0.033* | 2.42±0.042* |
|  | 100 | 1.75±0.085* | 1.90±0.054* | 1.88±0.036* | 2.59±0.038* |
| KMJ-10-1-4 | 0 | 1.00±0.039 | 1.00±0.025 | 1.00±0.209 | 1.00±0.025 |
|  | 25 | 1.14±0.036 | 1.38±0.037* | 1.19±0.036* | 1.46±0.034* |
|  | 50 | 1.42±0.057* | 1.69±0.030* | 1.28±0.026* | 1.70±0.039* |
|  | 100 | 1.64±0.071* | 2.01±0.041* | 1.54±0.053* | 2.34±0.050* |
| CR Dhan 601 | 0 | 1.00±0.042 | 1.00±0.039 | 1.00±0.051 | 1.00±0.019 |
|  | 25 | 1.18±0.037 | 1.74±0.038 | 1.50±0.064 | 1.38±0.024 |
|  | 50 | 1.54±0.069* | 2.14±0.049* | 1.93±0.062* | 2.24±0.007* |
|  | 100 | 1.92±0.088* | 2.63±0.086* | 1.99±0.062* | 2.65±0.020* |
| Tulsi Joha | 0 | 1.00±0.043 | 1.00±0.038 | 1.00±0.030 | 1.00±0.027 |
|  | 25 | 1.28±0.042* | 2.23±0.032* | 1.52±0.039* | 1.47±0.040* |
|  | 50 | 1.75±0.066* | 2.72±0.058* | 1.79±0.028* | 1.85±0.046* |
|  | 100 | 1.96±0.070* | 2.87±0.061* | 1.88±0.077* | 2.64±0.053* |
| Joymati | 0 | 1.00±0.021 | 1.00±0.080 | 1.00±0.033 | 1.00±0.023 |
|  | 25 | 1.53±0.044* | 2.26±0.074* | 2.00±0.047* | 1.92±0.032* |
|  | 50 | 1.73±0.046* | 2.36±0.076* | 2.38±0.053* | 2.59±0.035* |
|  | 100 | 2.05±0.061* | 2.88±0.070* | 2.55±0.050* | 2.91±0.052* |

Data presented are mean ± S.E.(n=10).

Significant mean difference between control and stress plants were significant at *P* < 0.05 (*) by Tukey test.
