# Supplementary material for: Morpho-physiological analysis of tolerance to aluminum toxicity in rice varieties of North East India
Source: PLoS One. 2017 Apr 27;12(4):e0176357. doi: 10.1371/journal.pone.0176357 (PMC5407633; doi:10.1371/journal.pone.0176357)
Supplement: S4 Table — (DOCX) [file pone.0176357.s004.docx]

**Table. S6.** Effect of Al treatment on MDA content in rice roots at 24 and 48h interval.

| **Varieties** | **Al Conc.(µM)** | **MDA (µmoles g^-1^FW)** | |
| --- | --- | --- | --- |
|  |  | **24h** | **48h** |
| Disang | 0 | 0.14±0.011 | 0.36±0.080 |
|  | 25 | 0.22±0.009 | 0.46±0.011 |
|  | 50 | 0.4070.010* | 0.53±0.030 |
|  | 100 | 0.48±0.033* | 0.56±0.108 |
| Swarna sub 1C | 0 | 0.19±0.027 | 0.44±0.079 |
|  | 25 | 0.44±0.039 | 0.63±0.024 |
|  | 50 | 0.5070.098* | 0.66±0.019 |
|  | 100 | 0.59±0.044* | 0.82±0.084* |
| Naveen | 0 | 0.22±0.050 | 0.38±0.029 |
|  | 25 | 0.36±0.020 | 0.82±0.072* |
|  | 50 | 0.55±0.012* | 0.92±0.030* |
|  | 100 | 0.64±0.045* | 0.99±0.038* |
| KMJ-6-1-1 | 0 | 0.45±0.016 | 0.43±0.056 |
|  | 25 | 0.55±0.027 | 0.62±0.025* |
|  | 50 | 0.71±0.040* | 0.74±0.035* |
|  | 100 | 0.92±0.059* | 1.07±0.040* |
| Tapaswini | 0 | 0.52±0.026 | 0.42±0.032 |
|  | 25 | 0.64±0.028 | 0.65±0.064 |
|  | 50 | 0.68±0.025* | 0.77±0.114* |
|  | 100 | 0.81±0.030* | 0.86±0.053* |
| Badsahbhog | 0 | 0.51±0.040 | 0.58±0.046 |
|  | 25 | 0.59±0.001 | 0.66±0.066 |
|  | 50 | 0.71±0.028* | 0.84±0.151 |
|  | 100 | 0.89±0.051* | 1.03±0.070* |
| Ranjit | 0 | 0.49±0.020 | 0.24±0.087 |
|  | 25 | 0.66±0.071 | 0.53±0.077 |
|  | 50 | 0.83±0.066* | 0.72±0.026* |
|  | 100 | 0.89±0.085* | 0.86±0.055* |
| Lachit | 0 | 0.57±0.022 | 0.43±0.021 |
|  | 25 | 0.78±0.079 | 0.76±0.170 |
|  | 50 | 0.81±0.038* | 0.99±0.037* |
|  | 100 | 0.93±0.037* | 1.18±0.092* |
| KMJ-6-1-2 | 0 | 0.29±0.038 | 0.57±0.206 |
|  | 25 | 0.35±0.050 | 1.06±0.008 |
|  | 50 | 0.42±0.029 | 1.16±0.018* |
|  | 100 | 0.60±0.021* | 1.26±0.101* |

Continued…..

| Aijung | 0 | 0.22±0.036 | 0.51±0.052 |
| --- | --- | --- | --- |
|  | 25 | 0.42±0.011* | 0.74±0.084 |
|  | 50 | 0.58±0.010* | 0.90±0.106* |
|  | 100 | 0.62±0.055* | 1.12±0.082* |
| Kola Joha | 0 | 0.15±0.012 | 0.52±0.055 |
|  | 25 | 0.33±0.014* | 0.89±0.087 |
|  | 50 | 0.43±0.015* | 0.97±0.142* |
|  | 100 | 0.63±0.021* | 1.08±0.062* |
| Sahbhagi Dhan | 0 | 0.21±0.015 | 0.43±0.022 |
|  | 25 | 0.57±0.034* | 0.54±0.011 |
|  | 50 | 0.72±0.016* | 0.64±0.041* |
|  | 100 | 0.75±0.020* | 0.77±0.029* |
| Cauveri | 0 | 0.16±0.008 | 0.44±0.062 |
|  | 25 | 0.48±0.030* | 0.87±0.18 |
|  | 50 | 0.54±0.010* | 1.01±0.072* |
|  | 100 | 0.65±0.022* | 1.14±0.079* |
| Gautam | 0 | 0.45±0.010 | 0.50±0.044 |
|  | 25 | 0.69±0.100 | 0.68±0.035 |
|  | 50 | 0.71±0.048* | 0.76±0.029* |
|  | 100 | 0.85±0.028* | 0.89±0.041* |
| Swarna | 0 | 0.16±0.019 | 0.61±0.107 |
|  | 25 | 0.45±0.080* | 0.79±0.023 |
|  | 50 | 0.52±0.049* | 0.97±0.055* |
|  | 100 | 0.77±0.060* | 1.05±0.064* |
| Kapilee | 0 | 0.37±0.076 | 0.33±0.042 |
|  | 25 | 0.44±0.020 | 0.62±0.037* |
|  | 50 | 0.68±0.067* | 0.90±0.055* |
|  | 100 | 0.75±0.068* | 1.12±0.093* |
| KMJ -2-1-4 | 0 | 0.40±0.022 | 0.48±0.077 |
|  | 25 | 0.6370.024 | 0.59±0.168 |
|  | 50 | 0.8270.019 | 0.87±0.014 |
|  | 100 | 0.8670.011* | 1.14±0.098* |
| Bahadur | 0 | 0.44±0.023 | 0.56±0.064 |
|  | 25 | 0.52±0.032 | 0.76±0.055 |
|  | 50 | 0.60±0.013* | 0.90±0.078* |
|  | 100 | 0.68±0.016* | 1.11±0.028* |
| Mashuri | 0 | 0.57±0.037 | 0.62±0.140 |
|  | 25 | 0.71±0.033 | 0.82±0.110 |
|  | 50 | 0.77±0.034 | 0.99±0.084 |
|  | 100 | 0.93±0.054* | 1.10 ±0.101 |

Continued…..

| Chandrama | 0 | 0.43±0.069 | 0.48±0.030 |
| --- | --- | --- | --- |
|  | 25 | 0.73±0.070 | 0.53±0.007 |
|  | 50 | 0.82±0.066* | 0.62±0.037* |
|  | 100 | 0.91±0.068* | 0.75±0.024* |
| KMJ-10-1-4 | 0 | 0.16±0.038 | 0.51±0.096 |
|  | 25 | 0.44±0.032* | 0.88±0.094* |
|  | 50 | 0.57±0.079* | 1.06±0.025* |
|  | 100 | 0.71±0.069* | 1.08±0.051* |
| CR Dhan 601 | 0 | 0.58±0.062 | 0.48±0.077 |
|  | 25 | 0.73±0.062 | 0.59±0.168 |
|  | 50 | 0.84±0.038* | 0.87±0.014 |
|  | 100 | 0.9470.022* | 1.22±0.098* |
| Tulsi Joha | 0 | 0.56±0.011 | 0.57±0.053 |
|  | 25 | 0.68±0.068 | 1.06±0.067 |
|  | 50 | 0.83±0.043 | 1.16±0.092 |
|  | 100 | 0.96±0.024 | 1.26±0.138 |
| Joymati | 0 | 0.33±0.051 | 0.55±0.087 |
|  | 25 | 0.60±0.030* | 0.79±0.043 |
|  | 50 | 0.89±0.043* | 1.04±0.08* |
|  | 100 | 0.99±0.054* | 1.27±0.074* |

Data presented are mean ± S.E.(n=10).

Significant mean difference between control and stress plants were significant at *P* < 0.05 (*) by Tukey test.
