# Supplementary material for: Morpho-physiological analysis of tolerance to aluminum toxicity in rice varieties of North East India
Source: PLoS One. 2017 Apr 27;12(4):e0176357. doi: 10.1371/journal.pone.0176357 (PMC5407633; doi:10.1371/journal.pone.0176357)
Supplement: S5 Table — (DOCX) [file pone.0176357.s005.docx]

**Table S7**. Classification of 24 rice genotypes based on root tolerance index (RTI) values at 48h.

| RTI value | Class | Rice varieties | No. of genotypes |
| --- | --- | --- | --- |
| > 0.90 | Tolerant | Disang, Swarna sub 1C,Naveen, Ranjit,Lachit,Tapaswini, KMJ-6-1-2,KMJ-6-1-1, Badsahbhog, Aijung | 10 |
| 0.80-0.90 | Moderately tolerant | Gautam,Swarna,KMJ-2-1-4,Bahadur,Kola Joha, Caveri, Kapilee, Sahbhagi Dhan | 8 |
| < 0.80 | Susceptible | Joymati,Chandrama, CR Dhan 601,Mashuri,KMJ-10-1-4,Tulsi Joha, | 6 |

Relative tolerance index among the 24 genotypes were observed at 48 h. Disang genotypes showed higher tolerance index compare another genotypes.
