# Supplementary figures and images for: Morpho-physiological analysis of tolerance to aluminum toxicity in rice varieties of North East India
Source: PLoS One. 2017 Apr 27;12(4):e0176357. doi: 10.1371/journal.pone.0176357 (PMC5407633; doi:10.1371/journal.pone.0176357)

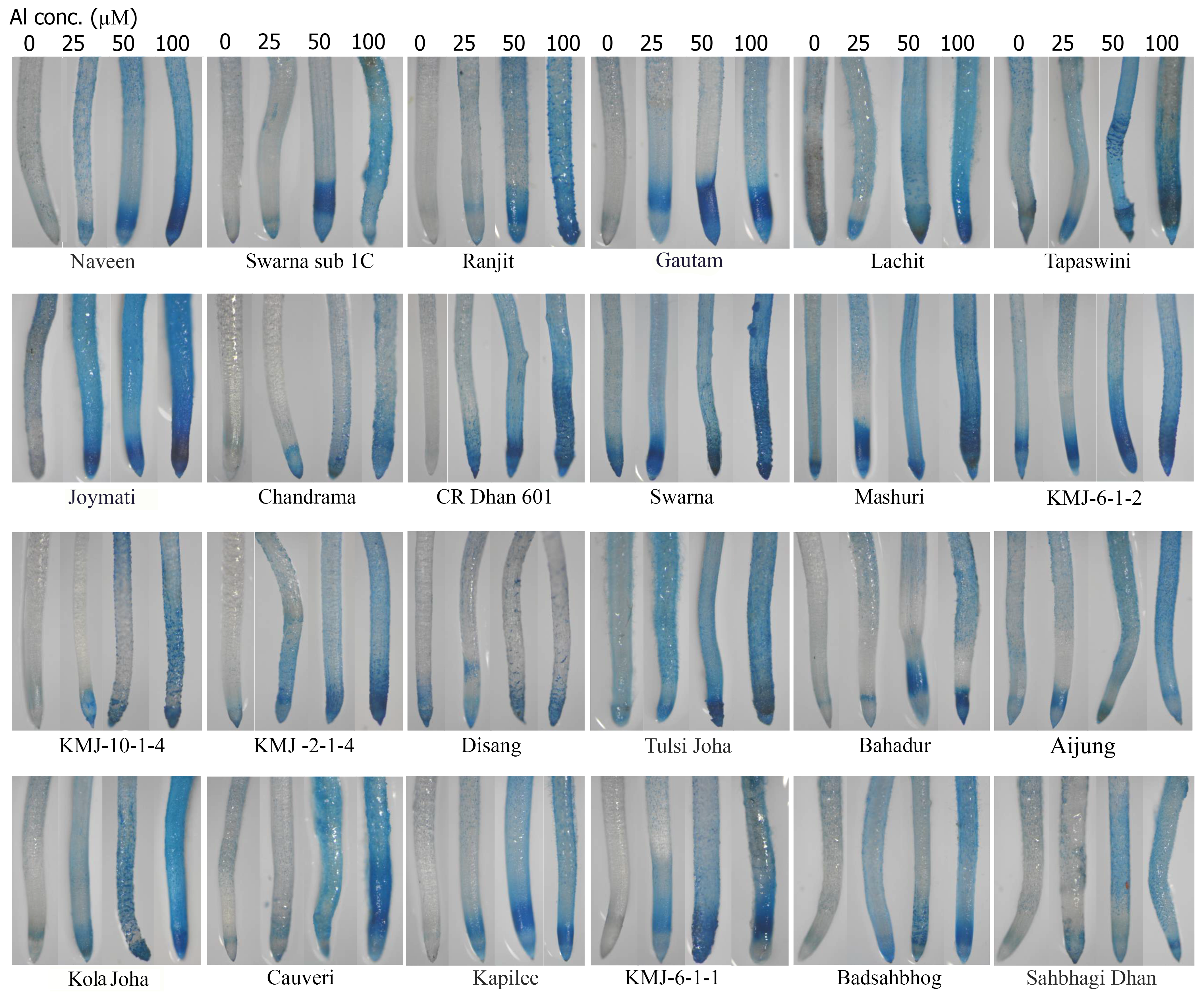

Supplement: S1 Fig — Microscopic views of Evan’s blue stained in root tips. Intense blue stained root portions represent increased Evans blue uptake at 48h. (TIF) [file pone.0176357.s006.tif]

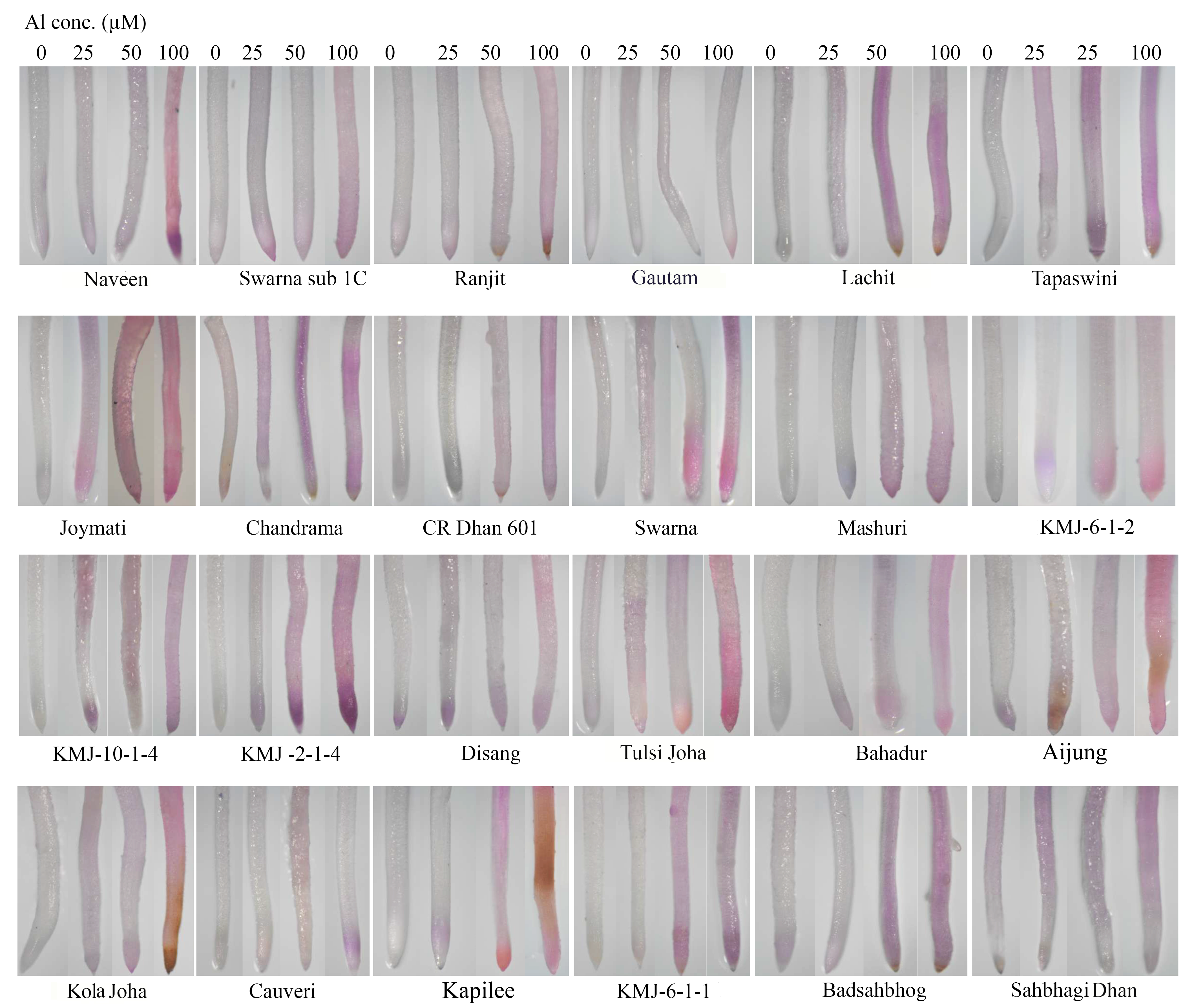

Supplement: S2 Fig — Microscopic views of Schiffs reagents stained of root tips. Intense pinkish colour represent lipid peroxidation by the root cells at 48 h. (TIF) [file pone.0176357.s007.tif]
